# Fine Brown Carbon Aerosol Emitted by Wood Pyrolysis - New Harmful Tracers and Cytotoxicity in Human Lung Cells

**DOI:** 10.1021/acs.chemrestox.6c00273

**Published:** 2026-10-01

**Authors:** Nadira Zendi, Vinh Nguyen, Bartłomiej Witkowski, Anna Pogorzelska, Małgorzata Milczarek, Tomasz Gierczak

**Affiliations:** † Faculty of Chemistry, 49605 University of Warsaw, al. Żwirki i Wigury 101, 02-089 Warsaw, Poland; ‡ Department of Biomedical Research, 49921 National Medicines Institute, 00-725 Warsaw, Poland

## Abstract

Biomass burning (BB) is a major global source of light-absorbing organic aerosols, the so-called atmospheric brown carbon (BrC), which negatively affects human health. However, the interplay between the molecular composition and toxicity of BB emissions remains ambiguous. In this study, cytotoxicity analyses and molecular-level characterization of fine BrC generated by wood pyrolysis at 350 °C, representative of smoldering combustion during vegetation fires, were performed. Solvent-extractable BrC was analyzed using UHPLC-ESI-ToF-MS coupled with a nontarget analysis (NTA) workflow, and cytotoxicity was evaluated on the A549 (CCL-185) human lung carcinoma cell line. In the dissolved BrC, 776–1132 unique molecular features were detected. The aqueous fraction was dominated by lignin-derived phenols and aromatic acids, whereas organic solvents (methanol and acetone) preferentially extracted less oxidized, more hydrophobic compounds, such as terpenoid- and sterol-like species, including several emerging BB tracers. Furthermore, reduction in metabolic activity, cellular membrane damage, death phenotype, and morphology were investigated. Cytotoxicity of solvent-extractable BrC increased in the order water < methanol < acetone. Descriptor-based screening showed that cytotoxicity was likely associated with lipophilic compounds capable of membrane partitioning and redox-active moieties, contributing to oxidative stress. The harmful BrC tracers identified included benzoic and cinnamic acids, methoxyphenols, quinols, and natural compounds such as flavonoids, coumarins, indoles, and plant sterols. The highest contributors to baseline narcosis were 3-hydroxybenzoic, salicylic, *p*-coumaric acids, benzene-1,2,4-triol, methylxanthoxylin, methyl ester of heteropeucenin, coniferylaldehyde, and agelasine. These results demonstrate that chemical composition strongly governs BrC toxicity. Moreover, by combining comprehensive molecular-level characterization with simple descriptors, it is possible to identify potentially harmful compounds within complex BB emissions. The presented approach provides a framework for identifying potentially harmful components of BrC aerosols and supports improved assessment of their health impacts.

## Graphical abstract

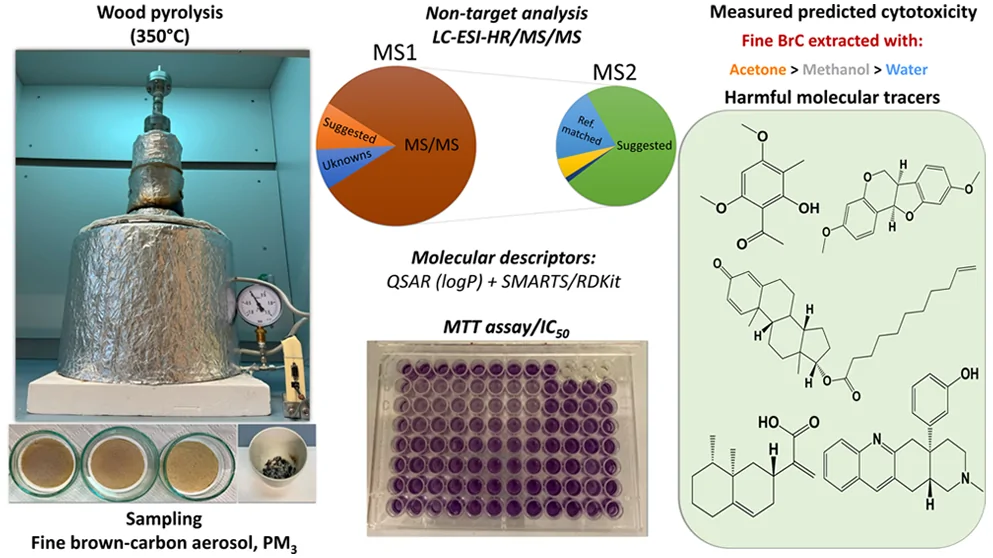

## 1 Introduction

Biomass is a nonfossil material derived primarily from plants and is widely used for household cooking, heating, and industrial energy production.^1,2^ In addition to domestic and industrial uses, vegetation fires are major contributors to open, uncontrolled biomass burning (BB).^3^ Every year, open BB harms infrastructure, ecosystems, and livelihoods of millions of people around the globe.^3^ Moreover, BB releases fine particulate matter (PM), greenhouse gases, black carbon, and other climate-active species.^4,5^ BB is also the major source of brown carbon (BrC), which exhibits positive radiative forcing due to its strong light absorption in the near-UV and visible regions.^6,7^ These species contribute to the increase in global temperatures, which induces more favorable fire-weather conditions.^8^ As such, wildfire intensity and associated emissions continue to increase, with extreme events now occurring worldwide, affecting vulnerable ecosystems.^8,9^

Following more widespread and severe fire events, BB pollutants can adversely affect public health even far from emission sources, because smoke plumes can be transported over hundreds to thousands of kilometers.^10^ Global estimates for 2010–2019 indicate that more than 2 billion people per year were exposed to at least 1 day of substantial air pollution originating from landscape fires, primarily PM_2.5_ and ozone.^11^ Exposure to fire-related pollution can therefore affect populations far larger than those directly exposed to flames and heat.^11^ Indeed, earlier global modeling studies estimated that exposure to landscape-fire smoke contributes to 0.34–0.68 million premature deaths annually.^12,13^ Exposure to BB emissions was associated with respiratory and cardiovascular conditions, and increased morbidity.^11,14^ The specific conditions induced by BB pollutants include asthma, lung cancer, and poor cognitive performance.^11,15^ At the cellular level, fine PM emitted or influenced by BB can induce oxidative stress (OS), inflammation, mitochondrial dysfunction, DNA damage, apoptosis, and impairment of the epithelial barrier.^16,17^ These adverse health effects have been associated with the chemical composition of PM emitted by BB.^17–20^ However, data sets combining comprehensive molecular characterization of BB aerosols with experimental toxicological analyses remain scarce.^21–23^

The composition of BrC is extremely complex and evolves during atmospheric processing.^6,24–26^ Organic carbon emitted by BB contains thousands of unique organics, including aliphatic and aromatic species, terpenoids, resin acids, and products of thermal degradation and biological origin.^6,21,24^ ^,^ ^25,27^ At the same time, a large fraction of BB pollutants remains unidentified, thus lacking toxicological characterization,^24,25,28^ thus falling outside existing regulatory inventories.^29,30^ Consequently, molecular-level characterization of BB tracers is important from a public health and regulatory perspective, enabling better hazard-based risk management,^31,32^ as current air-quality standards rely primarily on particle mass.^33^

This study aimed to analyze the interplay between molecular composition and cytotoxicity of fine BrC. Here, BrC was generated by wood pyrolysis close to ignition temperature to mimic the smoldering combustion during large vegetation fires.^34–37^ Furthermore, low-temperature pyrolysis of wood generates tar-ball proxies,^38^ which are among the major contributors to wildfire plumes.^36^ Moreover, significant differences in composition and properties of water- and organic-soluble fractions of BB aerosol and wood tar were reported.^19,39–41^ For this reason, in this work, BrC was extracted with water, methanol, and acetone to recover compounds over a broad polarity range.

Because fine particles, including PM_2_._5_, deposit efficiently in the alveolar region, A549 human lung epithelial cells are commonly used as an in vitro model of alveolar exposure to airborne pollutants.^42^ In the present study, the 3-(4,5-dimethylthiazol-2-yl)-2,5-diphenyltetrazolium bromide (MTT) and lactate dehydrogenase (LDH) assays were used to assess cellular metabolic activity and the extent of plasma-membrane damage, respectively.^42,43^ Additionally, staining-based microscopic imaging was used to evaluate changes in cell morphology, attachment, and monolayer integrity.^43^

Complementary to cytotoxicity analyses, the molecular composition of fine BrC was studied with ultrahigh-performance liquid chromatography coupled with electrospray ionization time-of-flight tandem mass spectrometry (UHPLC–ESI–ToF–MS/MS). Previous toxicological studies have often focused on volatile BB emissions detectable by gas chromatography (GC), particularly PAHs.^6,22,44^ Therefore, the presented work aimed to provide complementary information on the less-volatile, more-polar components of BrC.^24,25,32^ To this end, a nontarget analysis (NTA) workflow recently developed by our group was utilized to provide molecular-level characterization of fine BrC.^37^ While classical (targeted) protocols are largely limited to preselected standards,^30^ NTA can propose molecular structures of pollutants based solely on MS/MS data.^30,45^ For this reason, NTA has recently emerged as a powerful tool in identifying combustion-related pollutants.^21,25,46^

Moreover, properties of molecular BrC tracers identified via NTA related to toxicity were predicted, including octanol–water partition coefficients (logP) and structural alerts for ROS activity and electrophilic protein binding. Higher logP values indicate greater potential for membrane partitioning and baseline narcosis,^20,47^ whereas quinones, catechols, nitroaromatics, and related conjugated structures may promote OS through redox cycling.^20,36^ As such, redox-active BrC tracers were linked to mitochondrial dysfunction, glutathione depletion, and DNA damage in the A549 cell line.^48^ Electrophilic compounds may additionally disrupt cellular function through covalent protein binding.^36,49^ These descriptors were therefore used to identify BrC constituents likely contributing to the observed cytotoxic responses.

## 2 Experimental Section

Materials and reagents are listed in Section S1 of the Supporting Information (SI). All NTA results (section 2.3.1) and estimated toxicological end points (section 2.4) are available in the Repository for Open Data (RepOD).^50^

### 2.1 Brown Carbon Aerosol Generation and Sampling

Fine BrC (PM_3_) was generated and collected using a custom-built benchtop combustor.^37^ In all experiments, commercial wood pellets (see section S1) were used as a fuel. Pellets (25 g) were heated at 350 °C for 1.5 h, and the bath gas flow (N_2_) was 2 L min^–1^ in a 3 kW ceramic resistance heater. For the particle collection, three filters were used in parallel. First, the emissions were passed through a graded steel mesh filter (pore size from 500 to 50 μm) followed by two 47 mm filters. The first filter (hydrophobic PTFE, pore size 3 μm, Fluoropore, FSLW07400) was followed by a second PTFE-coated borosilicate glass microfiber filter (EMFAB TX40HI20-WW, Pallflex, Pall Life Sciences, aerosol retention 99.95%). The mass of fine BrC collected on the second (Pallflex) filter was determined using an analytical microbalance (Radwag, model XA 52.5Y). Before sampling, the second filter was placed in a desiccator for 24 h with dry molecular sieves; the mass of the collected PM_3_ was 30–40 mg. The filters were immediately extracted or stored at −80 °C.

For extraction, the filters were vortexed in 30 mL of water, methanol, or acetone for 30 min. Then, extracts were filtered through a 0.22 μm PTFE syringe filter and evaporated to dryness under reduced pressure using rotary evaporation (Labconco, model no. 7810033) at 30 °C or freeze-dried (aqueous extracts).^41^ The dried extracts were resuspended in dimethyl sulfoxide (DMSO) before cytotoxicity tests. The DMSO concentration in the cell medium was below 1% v/v.^51^

### 2.2 In Vitro Cytotoxicity Analyses of Solvent-Extractable BrC

#### 2.2.1 Cell Cultivation

A549 (CCL-185) lung carcinoma cell line was obtained from the American Type Culture Collection (ATCC, USA). Cells were grown in F-12k Nut Mix medium (Gibco Life Technologies, USA), supplemented with 10% fetal bovine serum (FBS, Biowest S181H-500, South America), 1% antibiotic solution (10,000 U mL^–1^ penicillin, and 10 mg mL^–1^ streptomycin, 25 μg mL^–1^ amphotericin B, Biowest), and 1% nonessential amino acids (10 mM) (Biowest, X0557). Cells were maintained in the humidified incubator at 37 °C in 5% CO_2_ (Thermo Scientific, USA). All cytotoxicity experiments were performed when the cells reached 80–90% confluence. Cell viability was determined by trypan blue solution staining using a Bio-Rad TC20 Automated Counter. Reduction and morphology were monitored using an optical microscope (Olympus DP10, Tokyo, Japan).

#### 2.2.2 Cell Metabolic Activity Reduction

The cytotoxicity analyses were performed using the MTT assay, which primarily reflects cell metabolic activity by detecting mitochondrial dehydrogenase activity.^52^ A549 cells were seeded in a 96-well plate at a density of approximately 120 × 10^3^ cells mL^–1^. After 24 h, increasing BrC concentrations (0–600 μg mL^–1^) were added to the cells and incubated for 24 h. Simultaneously, cells cultured in the absence of BrC served as controls. Wells containing only medium served as blanks and were used for background subtraction.

At the end of incubation, the medium was removed, and cells were washed with Dulbecco’s Phosphate-buffered saline (DPBS, Gibco Life Technologies). Next, 0.25 mg mL^–1^ MTT was added to each well and incubated for 3 h at 37 °C. The formed crystals were then dissolved in 0.1 mL of isopropanol. Absorbance was measured at 570 nm on a BioTek PowerWave XS 96-well microplate reader (Agilent, Santa Clara, USA). The metabolic activity was expressed by comparing the optical density (OD) at 570 nm between treated and control cells.

1 $$\%metabolicactivity=\frac{OD_{\mathrm{sample}}-OD_{\mathrm{blank}}}{OD_{\mathrm{control}}-OD_{\mathrm{blank}}}\times 100\%$$

In eq 1, OD_sample_ and OD_control_ are the absorbance values of treated cells and untreated cells (without PM_3_), respectively, and OD_blank_ is the absorbance of the medium without cells. Analyses were carried out in triplicate for each extraction solvent. IC_50_ represents the concentration of a compound required to inhibit 50% of metabolic activity. To calculate the half-maximal inhibitory concentration (IC_50_, μg mL^–1^) values for each solvent extract, the percentage inhibition of metabolic activity was first determined – eq 2.

2 %metabolic⁡activity⁡inhibition=100−%metabolic activity

IC_50_ values were calculated by fitting a dose–response inhibition curve via nonlinear regression based on a four-parameter logistic algorithm – eq 3.

3 $$Y=\min+\frac{\max-\min}{1+10^{\left(\log{IC}_{50}-X\right)\cdot Hillslope}}$$

In eq 3, Y is the response (% inhibition); X = logarithm of concentration; Min and Max = plateaus of the curve; Hill Slope = steepness of the curve. Data were plotted as average ± 2 standard deviation (σ) values. Each experiment gave an IC_50_ and an error value,^41^ and the results were expressed as average ± 3σ values. All the data were analyzed using two-way analysis of variance (ANOVA) with GraphPad Prism software (Version 8.0.2), followed by Dunnett’s, and Tukey’s post hoc tests for multiple comparisons among all fractions. Values with *p* < 0.05 were considered statistically significant.

#### 2.2.3 Cellular Membrane Integrity

Cytotoxicity was evaluated by measuring the release of LDH, an indicator of plasma membrane damage.^53^ LDH activity was quantified using a colorimetric CyQUANT LDH Cytotoxicity Assay Kit (Invitrogen, Thermo Fisher Scientific, Cat. No. C20301).

Following exposure of A549 cells to BrC, 50 μL of culture supernatant was collected and analyzed according to the manufacturer instructions. Untreated cells were used as the negative control (spontaneous release of LDH), while cells treated with 10 μL of 10X lysis buffer were used as the high control to quantify maximum LDH release. Absorbance was measured at 490 and 680 nm for background subtraction using the microplate reader (see section 2.2.2).

%Cytotoxicity was calculated as the percentage of LDH release relative to maximum LDH activity from three independent biological experiments – eq 4

4 $$\%Cytotoxicity=\frac{\left[\left(LDHactivityofcells\exp osedtoBrC\right)-\left(spon\tan eousLDHactivity\right)\right]}{\left[\left(\max imumLDHactivity-spon\tan eousLDHactivity\right)\right]}\times 100$$

Results were expressed as average ± 3σ values. All the data were analyzed using two-way analysis of variance (ANOVA) with GraphPad Prism software (Version 8.0.2), followed by Dunnett’s and Tukey’s post hoc tests for multiple comparisons among all fractions. Values with *p* < 0.05 were considered statistically significant.

#### 2.2.4 Assessment of Apoptosis, Cell Death, and Cellular Morphology

Cells exposed to the highest concentrations of PM_3_ extracts (600 μg mL^–1^) were gently rinsed and stained with the FITC Annexin V Apoptosis Detection Kit I (BD Pharmingen, San Jose, CA, USA, Cat. No. 556547). The staining solution was prepared by adding 100 μL of FITC Annexin V and 50 μL of Propidium Iodide (PI) per 1 mL of PBS. After 15 min of incubation in the dark, the FITC Annexin V (Channel 1, green, PMT: 587 V) and PI (Channel 2, red, PMT: 495 V) fluorescence signals were imaged using a Confocal Laser Scanning Microscope (Olympus, Shinjuku, Tokyo, Japan). FITC/PI filters were applied (488/520 nm and 543/600 nm), respectively. Living cells (Annexin V^–^/PI^–^), early (Annexin V^+^/PI^–^), and late apoptotic or necrotic cells (Annexin V^+^/PI^+^) were distinguished. Simultaneously, transmitted bright-field images (Channel 3) were acquired to assess cellular morphology.^54^

#### 2.2.5 Time-Kinetics Analysis

To evaluate the time-dependent kinetics of toxicity, cells were exposed to the highest concentration (600 μg mL^–1^) of BrC for 1, 3, and 24 h. After exposure, cells were washed, stained with Annexin V-FITC/PI solution, and confocal images were acquired as described in section 2.2.4. In parallel, metabolic activity inhibition and membrane integrity were investigated 3 and 24 h postexposure to PM_3_ (sections 2.2.2 and 2.2.3).

### 2.3 Liquid Chromatography Coupled with Mass Spectrometry

Analyses were conducted using a Shimadzu Q-TOF LCMS-9030 system equipped with an electrospray ionization (ESI) ion source.^37^ Analytes were separated with a Waters Acquity HSS-T3 column (100 × 2.1 mm, 1.8 μm) kept at 30 °C. The mobile phase consisted of 0.1% formic acid in water (A) and 0.1% formic acid in acetonitrile (B). The injection volume was 2 μL, and the flow rate of the mobile phase was 0.25 mL/min. The gradient elution program started at 5% B (0–10 min), increasing linearly to 25% (10–22 min), held for 13 min, then increased linearly to 95% (35–39 min), held for 5 min, then returned to 5% over 1 min and equilibrated for 15 min; the total runtime was 50 min.

The MS operated in positive and negative ionization modes, and the ToF resolution was approximately 45000. The flows of nebulizing, drying, and heating gases (N_2_) were 3.0, 10, and 10 L/min, respectively, with a 300 °C interface temperature. MS/MS spectra were acquired in data-dependent acquisition (DDA) mode with a 100–600 mass-to-charge ratio (*m*/*z*) precursor range (1s event time) and 40–600 *m*/*z* product scan (0.1 s event), three dependent events, 3000 intensity threshold, 1 s delay, and single charge state. Each sample was analyzed six times under both ionization modes using collision energy CE = 10 ± 5 V. Under these DDA conditions, ca. 4000 MS and MS/MS spectra were acquired.^25,37^

#### 2.3.1 Nontarget Screening and Feature Annotation

The molecular structures of BrC tracers were assigned using NTA with the workflow based on MS-DIAL (v5.3.240617) and MS-FINDER (v3.61).^37^ The workflow included raw data processing, spectra deconvolution, and assignment of molecular structures (feature annotation). First, the Shimadzu.lcd files containing MS and MS/MS spectra underwent mass-peak selection using the following parameters: MS tolerance = 0.01 Da, MS/MS tolerance = 0.025 Da, *m*/*z* range 100–600, maximum charge = 3, types of ions included [M-H]^−^, [2M-H]^−^, [M + H]^+^, [M+NH_4_]^+^, and [M + Na]^+^. Afterward, noise filtering and thresholding were performed: minimum peak height 5000(−) and 10000­(+), mass slice width: 0.1 Da, linear smoothing over 3–5 scans. Feature annotation was performed using internal (MS-DIAL) and external MS and MS/MS libraries - Table S2. Furthermore, an in silico MS/MS spectra predictor embedded in MS-FINDER was used to annotate features not identified with sufficient confidence by mass spectra libraries.

The confidence levels in NTA are 1 (molecular structure confirmed with an authentic standard), 2 (MS/MS spectra matched with database), 3 (suggested structure, partial library match), 4 (elemental formula assignment), and 5 (unknowns) – Figure S1.^55^ Hence, at levels 2 and 3, structural assignments obtained using NTA are tentative unless later confirmed by an authentic standard.

General properties derived for features annotated at level ≤ 4 included double-bond equivalents (DBE), carbon oxidation state (OS_c_), Kendrick mass, and Kendrick Mass Defect (KMD). The detected features were assigned to six general classes based on the H/C and O/C ratios: condensed hydrocarbons, carbohydrates, lignin, lipids, peptides, and tannin-like molecules (section S3).

#### 2.3.2 Semiquantitative Analyses

Semiquantitative analyses were carried out using ten surrogate standards (five for positive and negative ionization modes, Table S1) to estimate the concentrations of the molecular tracers of fine BrC in the filter extracts. First, calibration was performed in the concentration range 0.001 to 0.05 mg L^–1^, and response factors (RFs) were derived for each standard – eq 5.

5 $$RF_{Surrogate}=\frac{A_{Surrogate}}{C_{Surrogate}}$$

In eq 5, A_surrogate_ and C_surrogate_ are the chromatographic peak area and concentration (mg L^–1^) of a surrogate standard, respectively, and RF_surrogate_ is the response factor. The concentrations of fine BrC tracers were obtained with eq 6.

6 $$C_{BrC}\left(\frac{mg}{L}\right)=\frac{A_{BrC}}{{\mathrm{RF}}_{closestsurrogate}}$$

In eq 6, *C*_*BrC*_ and A_BrC_ are the estimated concentration and chromatographic peak area of a BrC tracer, respectively. *RF*_closest surrogate_ is the response factor of the surrogate standard with the retention time closest to the given BrC tracer. Note that the use of surrogate standards introduces inherent error.^56^ For this reason, a 30% uncertainty was assumed for all values derived with eq 6.

### 2.4 Toxicity Estimates

Descriptors (Table 1) related to cytotoxicity were derived for WSOCs corresponding to the 80% cumulative concentration in each extract (see section 2.3.1).

**Table 1:** Estimated Molecular Properties Related to Cytotoxicity

Property	Description	Mechanisms	ref.
Hydrophobicity (logP)	Proxy for membrane partitioning and baseline narcosis	Membrane leakage, bilayer disruption, mitochondrial membrane depolarization	^47,57^
Reactive oxygen species (ROS) activity	Presence of redox-active moieties: quinones, catechols, nitroaromatics, conjugated systems	Redox cycling, superoxide and H_2_O_2_ generation, OS, mitochondrial dysfunction, Nrf2 activation, DNA damage	^20^
Protein binding (electrophilicity)	Presence of electrophilic functional groups capable of covalent adduct formation	Michael addition to cysteine residues, enzyme inhibition, disruption of redox homeostasis, and inflammatory signaling	^49^

OPERA v2.9.2 QSAR suite was used to estimate hydrophobicity, expressed as logP.^58^ Note that in baseline toxicity rankings, nutrient-like molecules (e.g., fatty acids and sterols, Tables S6–S8) were excluded. Furthermore, BrC tracers were screened for structural features associated with redox cycling potential using SMARTS substructure matching in RDKit with a self-written Python script.^59^ The reactive oxygen species (ROS) alert panel flagged quinone-like structures, catechols, hydroquinones, nitroaromatics, peroxides, and α,β-unsaturated carbonyls. Separate screening for electrophilic moieties (e.g., α,β-unsaturated carbonyls, aldehydes, epoxides, anhydrides) was performed to obtain the protein-binding alerts.

The estimated descriptors were used to evaluate the concentration-weighted score of the whole extract (henceforth referred to as total cytotoxicity), expressed as the total score for each panel - eq 7.

7 $$Total,cytotoxicity=\overset{n}{\underset{i=1}{\sum }}\mathrm{logP or alert score}\times \mathrm{concentration}\left(\frac{\mathrm{mg}}{L}\right)$$

In addition to estimating total cytotoxicity, contributions for individual BrC tracers were expressed as the normalized score for each screening panel - eq 8.

8 $$Contribution_{molecule}\left(\%\right)=\frac{\mathrm{logP}/\mathrm{alert score}\times \mathrm{concentration}\left(\frac{\mathrm{mg}}{L}\right)}{\overset{n}{\underset{i=1}{\sum }}\mathrm{logP}/\mathrm{alert score}\times \mathrm{concentration}\left(\frac{\mathrm{mg}}{L}\right)}$$

In eq 8, the percentage contributions to the predicted cytotoxicity are estimated using logP values, ROS and protein-binding scores, each expressed as the sum of structural alerts for the individual BrC tracers. Using this approach, the major molecular contributors in each panel were identified.

## 3 Results and Discussion

### 3.1 General Characteristics of Fine BrC

The number of features annotated with NTA at level ≤ 4 (Figure S1) was 989 (water), 611 (methanol), and 756 (acetone), enabling a general classification of the BrC tracers – Figure 1.^21,24,25^

**Figure 1:**
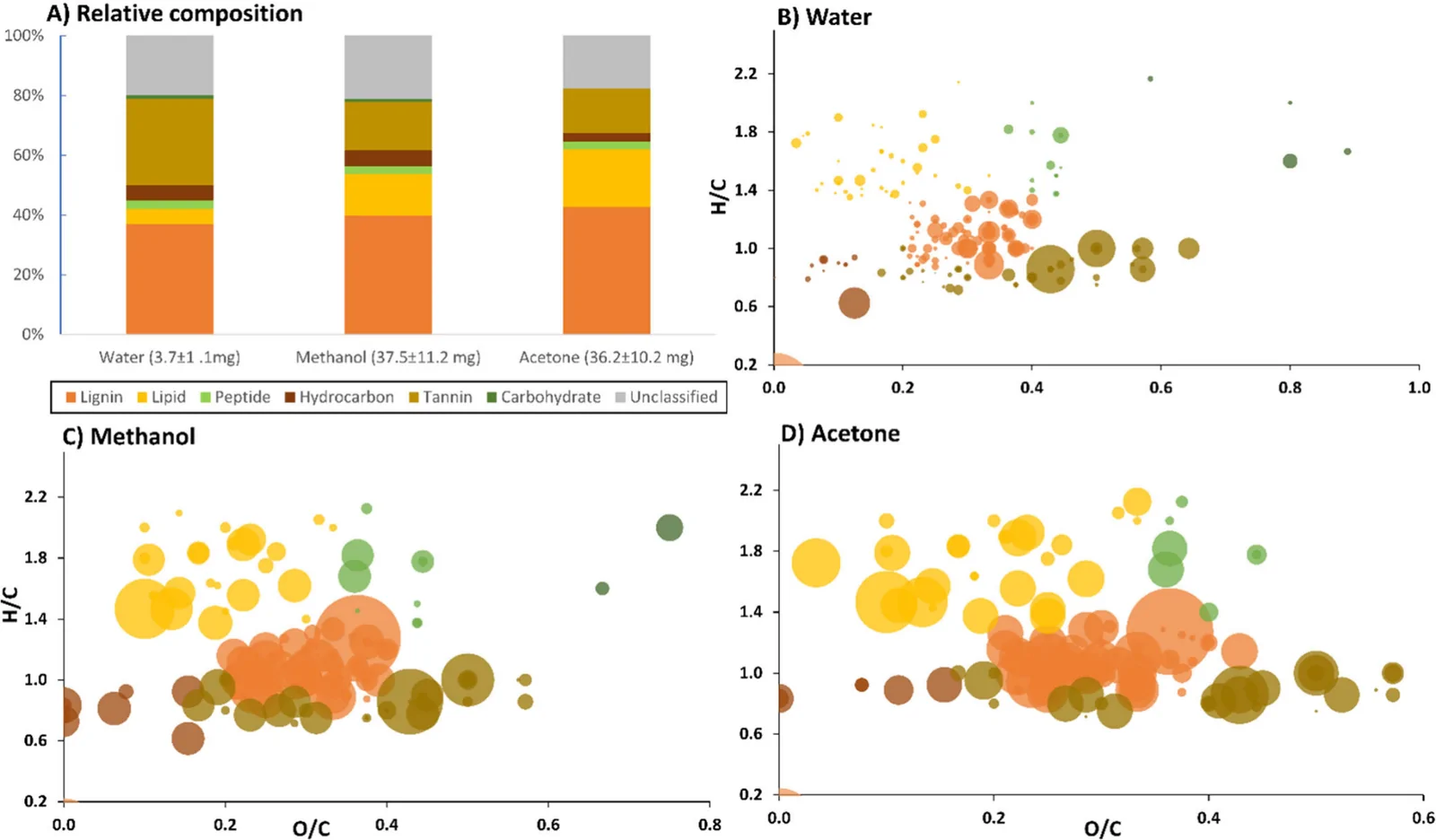
(A) Relative compositions of tracers identified at levels 1–5; note that elemental formula assignment is not necessary for semiquantitative analysis using chromatographic peak area (section 2.3). Amounts in mg are the mass leftover after drying (section 2.1). (B-D) Van Krevelen (VK) diagrams of fine BrC extracted with (B) water, (C) methanol, and (D) acetone. VK diagrams were derived for features annotated at level ≤ 4 (see sections 2.3 and S4). In (B-D), circle sizes correspond to the concentrations in the filter extract measured with surrogate standards (section 2.3).

The amount of BrC extracted with water was ten times lower compared with organic solvents (Figure 1A).^39,40^ In all extracts (Figure 1A), products of lignin pyrolysis constitute the single largest fraction, followed by tannin-like molecules.^46,60^ Relative to water, extraction with organic solvents increased the amount of lignins and lipids (particularly in acetone), while noticeably lowering the contribution of tannins and peptides.^19,60^ This shows an enrichment in less polar, more reduced organics in the order of water < methanol < acetone.^6,19,60^

The aqueous extract exhibits a broader O/C range, consistent with a higher recovery of more polar molecules - Figure 1B.^19,40,61^ On the other hand, methanol and particularly acetone extracts are enriched in less oxidized, reduced elemental formulas in the lignin- and lipid-like regions (Figure 1C and D).^40,60,61^ The VK diagrams (Figure 1B-D) also revealed a shift in the median O/C from 0.30 to 0.26 with increasing solvent strength, and an increase in reduced, lipid-like formulas (H/C > 1.5). Likewise, the solvent-dependent VK shifts (Figures 1B-D) are consistent with the less oxidized BrC extracted by organic solvents.^40,60,61^

Additional bulk properties of BrC were derived using the elemental formulas extracted with NTA – Figure 2.

**Figure 2:**
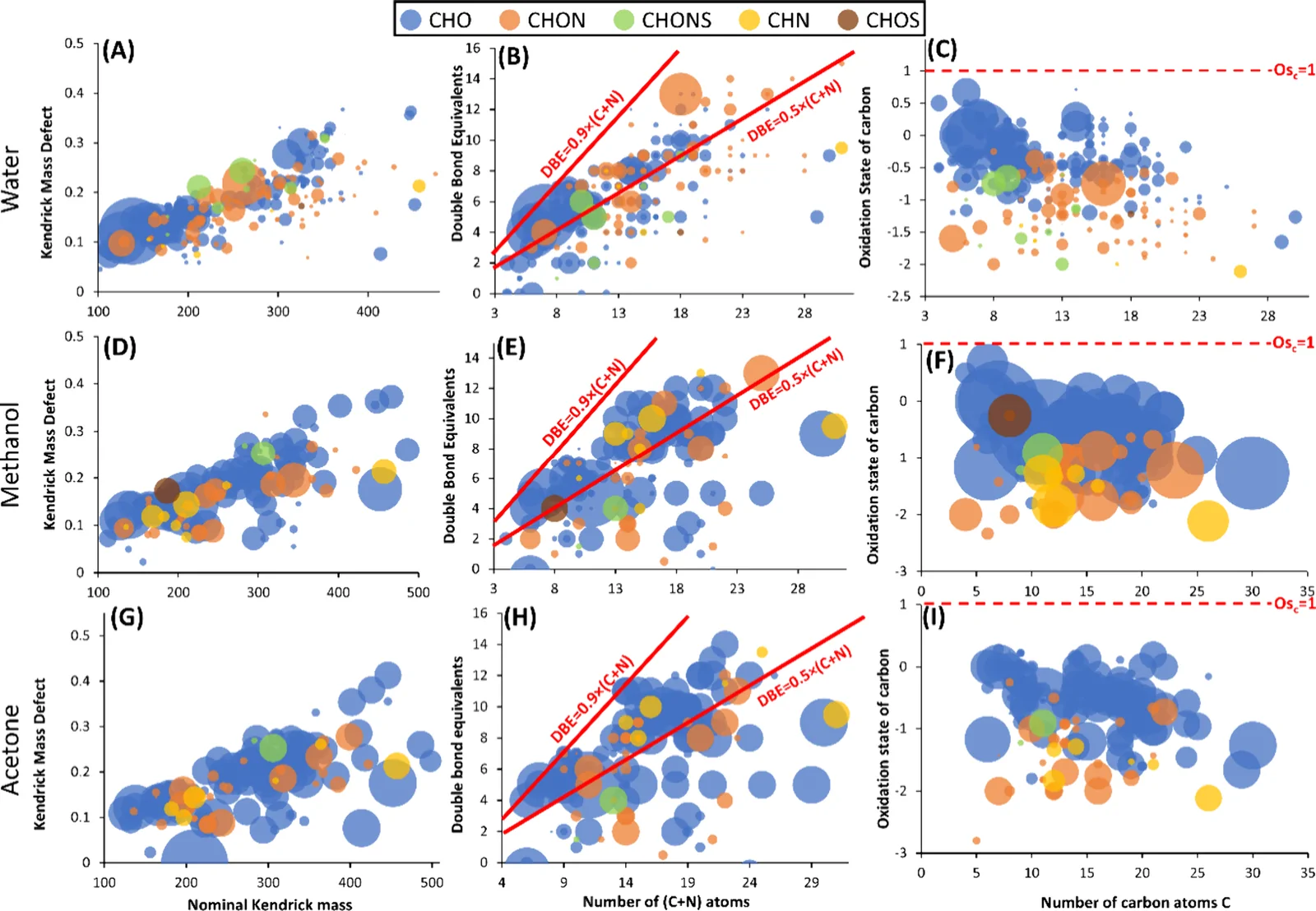
A, D and G: KMD plots. B, E and H: number (C+N) atoms vs DBE. C, F and I oxidation state (OS_c_) vs number of carbon atoms – see section S4 for derivation of these parameters. In B, E, and H, the potential chromophores (BrC space) are located in the areas between lines: DBE/(C+N) = 0.5 and 0.9. Circle sizes correspond to the concentrations of molecules composed of elements listed in the inset. Diagrams were derived for features annotated at level ≤ 4 (see sections 2.3 and S4).

In Figure 2, CHO formulas are dominant in all solvents, indicating the high abundance of lignins and tannins.^6,60,61^ Furthermore, water-soluble BrC (Figures 2A-C) showed a higher relative abundance of CHON formulas, likely corresponding to polar nitroaromatics, amines, and amides.^61,62^ Acetone extracts (Figure 2G-I) exhibited a higher relative proportion of CHO and hydrocarbon-like formulas, characteristic of less polar, more reduced molecules.^39,61,63^ Methanol (Figure 2 D, E and F) showed an intermediate selectivity, extracting a broad mixture of CHO and CHON species with a much smaller contribution from sulfur-containing classes (CHOS, CHONS), minor in all extracts.^24,61,64^

Plots in Figure 2A, D and G revealed a dominant, continuous cluster, indicative of overlapping homologous series of diverse BB tracers, with increasing KMD values for higher-molecular-weight (HMW) molecules.^24,65,66^ Furthermore, organic extracts (Figures 2D and G) contained a similar amount of less polar, HMW compounds.^24,61,62^ In Figures 2B, E and H, a clear correlation between DBE and (C+N) values can be observed, consistent with substituted benzenes and polyaromatics, and some fullerene-like hydrocarbons.^62^ The composition of organic extracts extends toward higher DBE values, indicative of less polar molecules,^19,40,61^ falling in the potential BrC region (Figure 2E and H).^60,62^ Figure 2C, F and I show clustering in the moderately reduced range, with mostly negative OS_c_ values, indicating more reduced, hydrocarbon-like structures consistent with partially oxidized aromatics and lignins.^60^ The absence of more positive OS_c_ values in organic solvents reflects limited oxidative processing, consistent with hydrophobic BrC.^60,67^ The aqueous extract shows a slight shift toward less negative OS_c_ values (Figure 2C), indicative of enrichment in more oxygenated, polar molecules (see also Figure 1B).^60,61^ Hence, the bulk molecular properties (Figures 1 and 2) of BrC generated in this work are representative of unaged, tar-like material emitted by pyrolysis of woody biomass.^24,60,62^

### 3.2 *In Vitro* Cytotoxicity of Solvent-Extractable BrC in A549 Cells

#### 3.2.1 Metabolic Activity and Membrane Damage

Results obtained with the MTT assay confirmed a reduction in cellular metabolic activity with increasing BrC concentration — Figure 3A.

**Figure 3:**
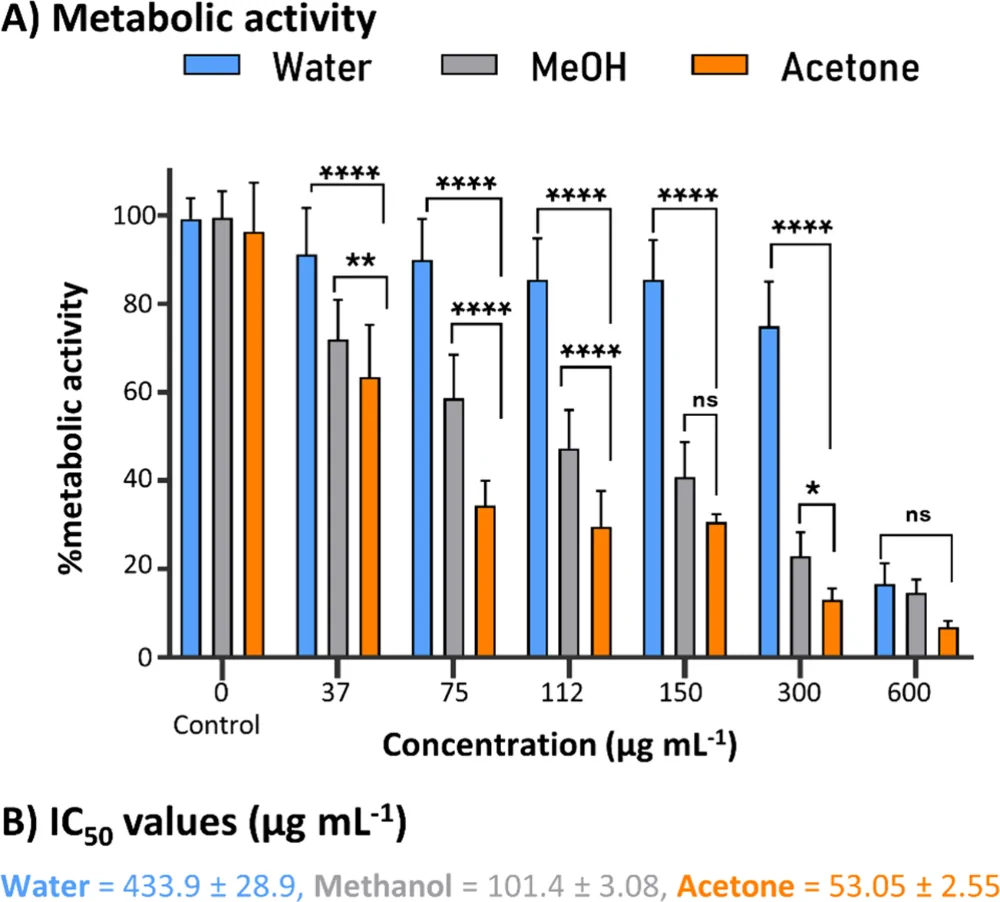
(A) A549 cell metabolic activity (eq 1) following exposure to solvent-extractable BrC, measured with the MTT assay. (B) IC_50_ (μg mL^–1^) values derived with eq 3. Lower IC_50_ values correspond to higher cytotoxicity. Statistical analyses of the MTT data (Tables S13 and S14) confirmed significant differences between organic and aqueous BrC fractions. (A) Brackets represent Tukey comparisons among solvent fractions at each concentration. Statistical significance notation: * and ** — *p* < 0.05, **** — *p* < 0.0001, and ns — *p > 0.05*.

The obtained IC_50_ values indicate that toxicity increased in the order water < methanol < acetone (Figure 3B). At BrC concentration = 300 μg mL^–1^, organic fractions and aqueous inhibited cell metabolic activity by >70% and <30%, respectively (Figure 3A). At 600 μg mL^–1^, inhibition of cell metabolic activity exceeded 80% for all solvents.

Comparison of the obtained IC_50_ values (Figure 3B) with the previously reported data is not straightforward because in vitro PM cytotoxicity assays are not fully standardized and the available cytotoxicity data also differ in particle source, preparation, methodology, and measured end point.^42,43^ Nevertheless, the cytotoxic potency of organic BrC extracts (Figure 3B) is comparable with that of laboratory and ambient PM influenced or emitted by combustion.^68–72^ Specifically, PM_2.5_ from straw- and wood-pellet combustion yielded a lethal concentration 50 (LC_50_) value of 40–130 μg mL^–1^ in A549 cells.^71^ Furthermore, a half-maximal effective concentration (EC_50_) of 200 μg mL^–1^ was reported for water-soluble particles generated during flaming, smoldering, and pyrolysis.^70^ Moreover, an IC_50_ = 117 μg mL^–1^ was reported for the 16HBE epithelial cell line for wintertime, urban PM_2.5_.^68^ For other urban PM_2.5_, an IC_50_ of approximately 310 μg mL^–1^ can be estimated from the published data.^41^ Finally, the IC_50_ values obtained in this work for organic extracts (Figure 3B) are within the range of EC_50_ = 150 μg mL^–1^ determined for NIST urban particulate matter SRM 1648a in A549 cells.^73^

However, the inhibition of metabolic activity did not necessarily coincide with equivalent membrane damage. For this reason, the release of LDH by A549 cells, indicative of a different degree of plasma membrane damage by organic and aqueous BrC fractions, was analyzed – Figure 4.

**Figure 4:**
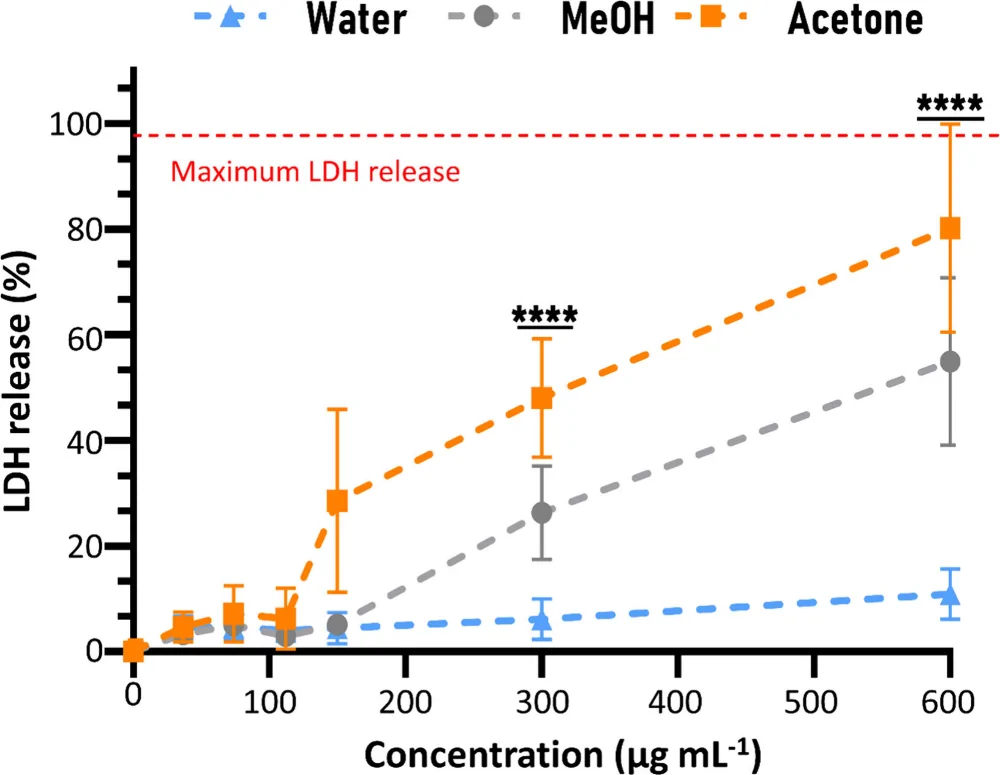
Relative cytotoxicity in A549 cells following 24 h exposure to dissolved BrC, measured with the LDH assay. Analysis of LDH data (Tables S15 and S16) confirmed that the differences in LDH activity between the aqueous and organic fractions were statistically significant at the two highest concentrations. Statistical significance notation: **** — *p* < 0.001.

A significant LDH release was observed from 150 μg mL^–1^ for acetone, from 300 μg mL^–1^ for methanol, and at 600 μg mL^–1^ for the aqueous fractions (Figure 4). At BrC concentration = 300 μg mL^–1^, the cytotoxicity induced by acetone, methanol, and aqueous extracts was 50, 26, and 6%, respectively. Increasing the concentration from 300 to 600 μg mL^–1^ resulted in an approximately 2-fold increase in LDH release for each fraction (Figure 4). Thus, the organic fractions caused substantially greater plasma-membrane damage than the aqueous fraction.^53,74^

While several studies reported significant increases of LDH release upon exposure to combustion-derived PM, others report no measurable release despite confirmed cytotoxicity.^69,75^ Nevertheless, the data obtained in this work are consistent with those reported for combustion-related PM in A549 and macrophage models.^76–78^ As such, wood smoke particles induced a concentration-dependent LDH release in RAW264.7 of 78–165%.^76^ Furthermore, PM_2.5_ emitted by crop residue increased membrane leakage and decreased viability of A549 cells at 150 μg mL^–1^.^77^ Moreover, an EC_50_ of 82–88 μg mL^–1^ based on LDH measurements was reported for traditional biomass fuel particles in BEAS-2B cells.^79^

Together, the MTT and LDH data indicate increasing severity of cellular injury in the order: water < methanol < acetone, and also show that metabolic impairment can precede substantial membrane disruption, particularly in the aqueous fraction. Moreover, despite methodological differences, the cytotoxic potency of the organic BrC fractions was within the same order of magnitude as reported 50% effect concentrations for combustion-derived PM and urban PM SRM.

#### 3.2.2 Cell-Death Phenotype and Cellular Morphology

To determine the mode of cell death induced by the BrC extracts, A549 cells were exposed to 600 μg mL^–1^ for 24 h, double-stained with Annexin V-FITC/PI solution - Figure 5.

**Figure 5:**
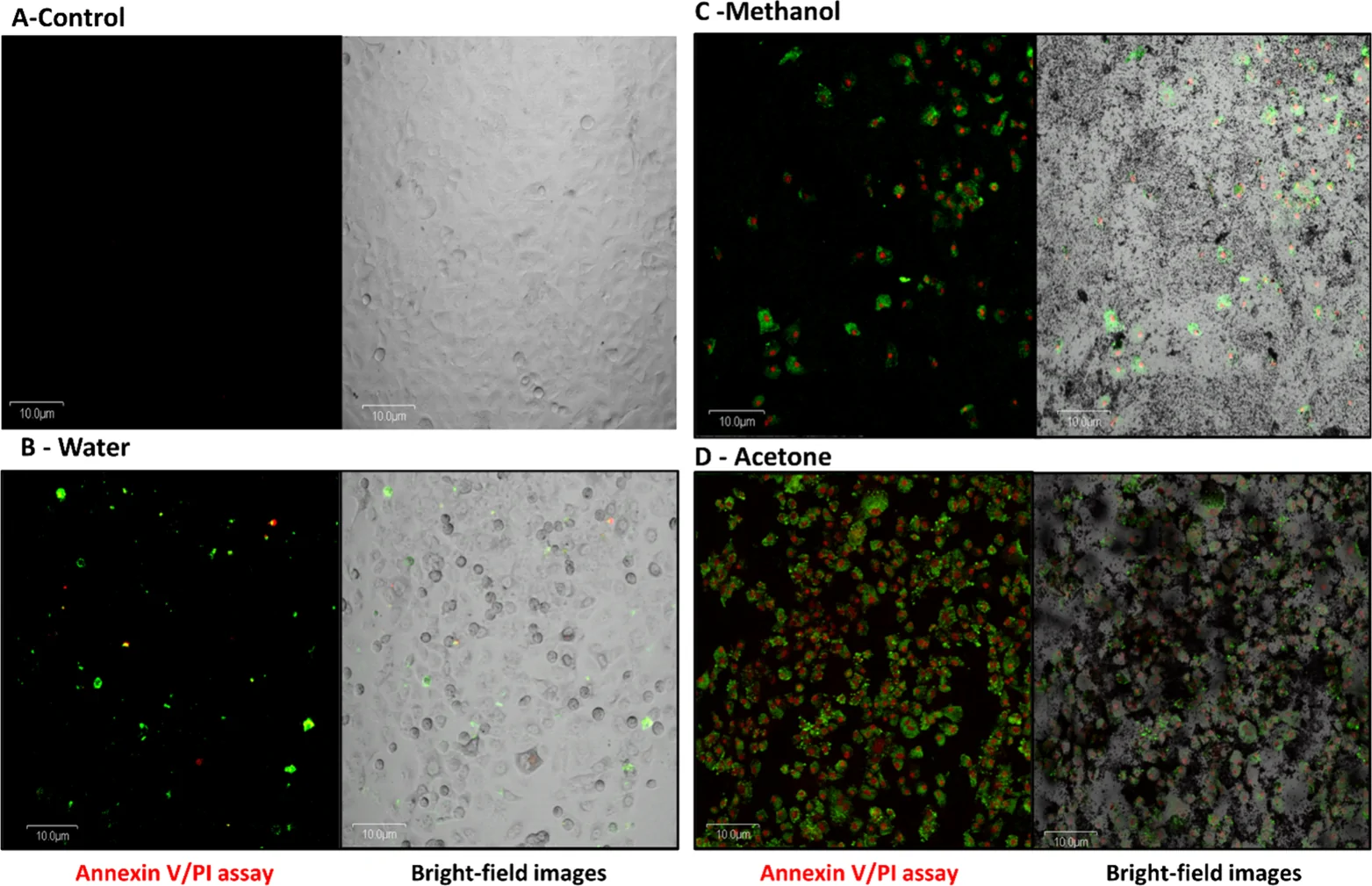
(A) Fluorescence and bright-field images of A549 cells: control cells. (B-D) Cells after 24 h exposure to BrC extracts at 600 μg mL^–1^. In the Annexin V/PI assay, early (Annexin V^+^/PI^–^ green) and late apoptotic or necrotic cells (Annexin V^+^/PI^+^, green and red) are distinguished from living cells (Annexin V^–^/PI^–^, no fluorescence signals).

In Figure 5A, control cells formed an intact epithelial monolayer with typical morphology and showed no Annexin V or PI fluorescence signals.^36,41,80^ In Figure 5B, A549 cells exposed to the aqueous extract remained largely attached and showed some Annexin V^+^/PI^–^ staining, consistent with an early apoptotic phenotype with preserved plasma-membrane integrity.^54,81^ On the other hand, A549 cells exposed to organic extracts exhibited indicators of cytotoxic stress (Figures 5C and D), particularly altered shape, detachment, and reduced monolayer integrity.^36,41^ Moreover, the organic fractions produced substantially greater cell detachment, loss of monolayer integrity, and Annexin V^+^/PI^+^ staining (Figure 5C and D), indicative of advanced stages of membrane-compromised injury.^82^ Hence, these observations (Figure 5) complement results presented in Figures 3 and 4, indicating that the aqueous fraction predominantly produced an early apoptotic phenotype with limited cellular membrane damage, whereas the organic fractions, particularly acetone, caused more advanced injury.^83,84^

#### 3.2.3 Time-Dependent Cytotoxicity in A549 Cells

Time-course analyses were conducted at the highest BrC concentration (600 μg mL^–1^) to determine whether the cytotoxicity was rapid or gradual — Figure 6.

**Figure 6:**
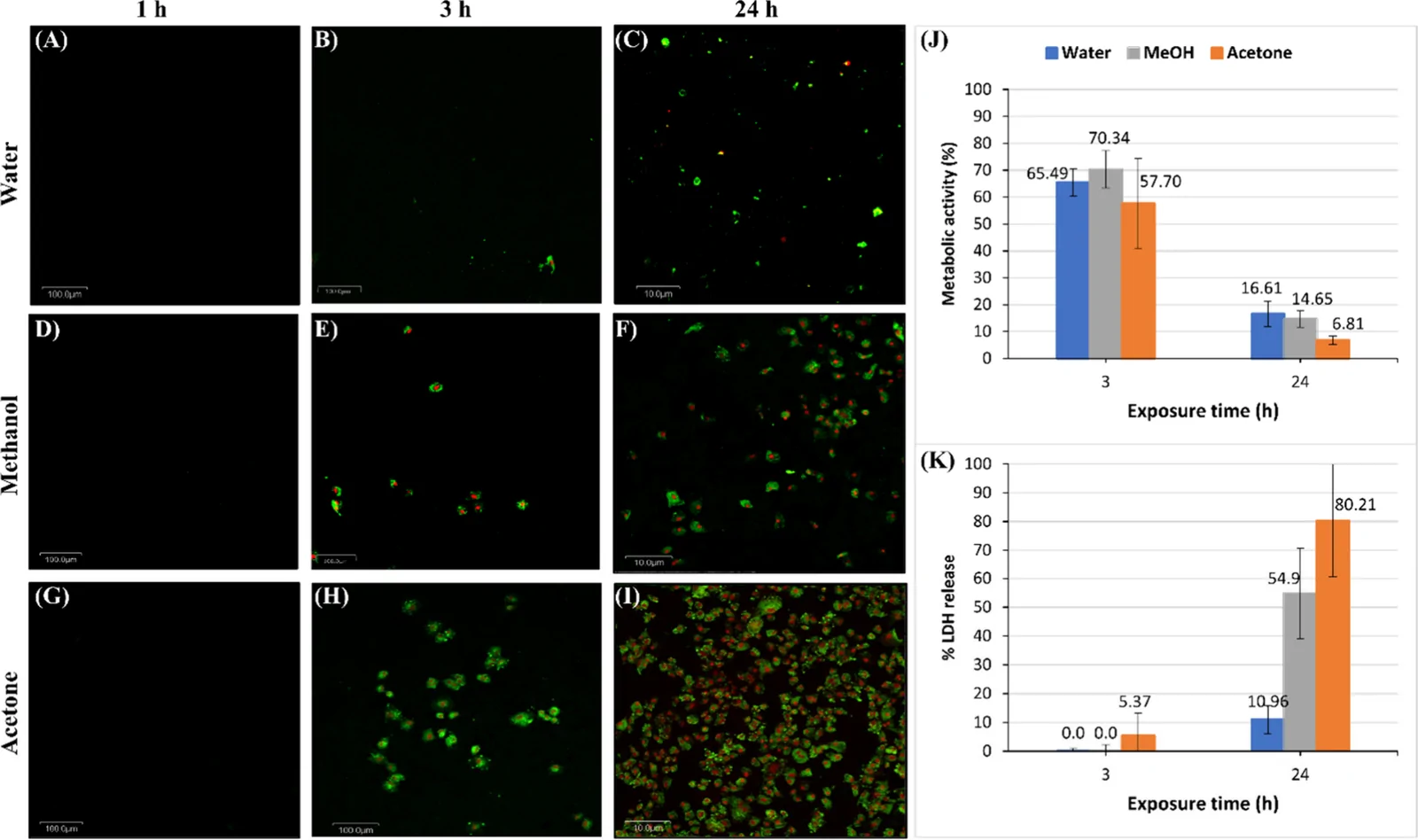
Annexin V/PI fluorescence images after 1, 3, and 24 h of exposure to 600 μg mL^–1^ of BrC dissolved in water (A-C), methanol (D-F), and acetone (G-I). Fluorescence microscopy images were acquired using FITC Annexin-V and red PI staining. Results obtained with the MTT (J) and LDH (K) assays after 3 and 24 h of exposure.

For all extracts, no detectable Annexin V or PI fluorescence was observed after 1 h while clear cytotoxic responses developed within 3 h and progressed thereafter through 24 h (Figure 6). The aqueous fraction predominantly induced green Annexin V^+^/PI^–^ staining (Figure 6A-C) with reduced metabolic activity (Figure 6J) but minimal early LDH release, noticeable only after 24 h (Figure 6K), indicating an early apoptotic phenotype with preserved membrane integrity. The methanol fraction showed metabolic inhibition and Annexin V^+^/PI^+^ signals (Figure 6D-F) after 3 h, with no bulk LDH release (Figure 6J,K). The acetone fraction caused marked metabolic inhibition alongside minor LDH release (Figure 6J,K), together with Annexin V^+^/PI^+^ staining (Figure 6H–I), indicating earlier progression toward extensive membrane-compromised cellular injury.^85–87^ Although early metabolic inhibition was comparable among the fractions, the organic fractions subsequently progressed toward greater membrane-compromised cellular injury, most prominently for the acetone fraction after 24 h of exposure.

#### 3.2.4 Comparison with Previous Data on Toxicity of Organic and Aqueous Fractions of BrC

To date, no studies have compared the cytotoxicity of aqueous and organic fractions of fine BrC generated by pyrolysis of wood close to ignition temperature (350 °C). At the same time, the available data indicate that cellular responses largely depend on extraction solvent and source characteristics (temperature and oxygen availability), which strongly influence the composition of combustion-related PM.^19,21,41,88^ Particularly, BrC extracted with dichloromethane and generated at 670 °C was more cytotoxic than that produced at 1035 °C.^88^ The higher toxicity of the lower-temperature BrC was attributed to more bioavailable, smaller molecular constituents, whereas higher-temperature combustion promoted molecular growth and carbonization.^88^

A previous study of tar-ball proxies generated by pyrolysis at 550 °C found that their water-soluble fraction was more cytotoxic during shorter exposure, whereas aqueous and organic fractions caused comparable cell death at the highest concentration after 24 h.^19^ The water-soluble fraction of the previously studied tar-balls induced stronger OS and lipid peroxidation, while the organic fraction caused greater superoxide production, mitochondrial membrane-potential loss, and DNA damage.^19^ In contrast, the aqueous fraction of BrC generated in this study at 350 °C (section 2.1) was consistently less cytotoxic than organic fractions, likely due to their higher lipophilicity. These data may also suggest a smaller contribution of ROS-active water-soluble constituents generated by wood pyrolysis at 350 °C than at 550 °C.^89,90^ As such, lignin decomposition at higher temperatures (550 °C) can produce more redox-active compounds, such as catechols and condensed aromatics, thus enhancing the ability of such BrC to induce oxidative stress and damage.^89,90^

### 3.3 Molecular Composition of Fine BrC

The number of BrC tracers with assigned molecular structure (NTA levels 1–3) ranges from approximately 300 to 900, representing between 41 and 71% of the total extracted features - Figure S1.^50^

In all extracts, the assigned molecular structures were primarily lignin-derived phenols, such as hydroxybenzoic and hydroxycinnamic acids, methoxyphenols, and aromatic carbonyls.^6,50^ Moreover, the most abundant compounds (Tables S6–S8) are consistent with the common BrC tracers, including hydroxyquinol,^25^ coumaric,^69^ and cinnamic acids,^60^ pyrogallol,^60^ salicylic acid,^65^ quinolines,^46^ terpenoids,^46^ and their derivatives. Several flavonoid-like structures were annotated, indicating the presence of plant secondary metabolites and phenols formed during pyrolysis.^60,91^ Furthermore, chromones and benzofurans were detected, representing more conjugated, oxygenated aromatics associated with BrC chromophores (Figures 2B, E, and H).^6,23^ A smaller fraction of nitrogen-containing aromatics (Figure 2), primarily quinoline-like structures, is also present in all extracts.^37,61^

The aqueous fraction was dominated by low-molecular-weight (LMW), oxygenated aromatic acids and phenols, including 3-hydroxybenzoic and 2,3-dihydroxybenzoic acid,^22,60^ and p-coumaric acid,^6,60^ hydroxyquinol,^46^ and coniferyl aldehyde - Table S6.^92^ Methanol-soluble BrC exhibited a broader compositional range, recovering moderately hydrophobic aromatics, such as methylxanthoxylin or pyrogallol.^6,22^ Some seldom-reported BrC tracers were also tentatively identified in the methanol extract, including 3,9-dimethoxypterocarpan, phenylquinoline, and heteropeucenin derivatives.^37^

In addition to more common BrC tracers, such as salicylic and coumaric acids,^6,93^ and pyrogallol,^60^ the acetone extract was enriched in lipids, terpenoids, and sterol-like species,^22,46^ annotated as boldenone undecylenate, triethylphosphate,^39,40^ confirming preferential extraction of more hydrophobic molecules.^39,61,63^ As such, steroids, sterols, and organophosphates were annotated among the most abundant compounds, especially in the acetone extract (Table S8). The NTA results strongly depend on the workflow,^94^ particularly the MS databases (Table S2),^37^ and structural assignments at levels 2 and 3 (section 2.3.1) are not unambiguous.^30^ Consequently, some ambiguous annotations (here, pharmaceuticals, steroid esters, or organophosphates) in Tables S6–S8 often result from low spectral match scores and can indicate the presence of molecules with similar structural motifs.^95^ The obtained structural assignments were analyzed to identify harmful molecular tracers in BrC extracts.

### 3.4 Harmful Molecular Tracers of Fine BrC Aerosol

Total and molecule-specific cytotoxicities were predicted for the BrC tracers annotated with NTA (section 2.4). All NTA annotations, logP values, and structural alerts are provided in Appendix 1.^50^ The predicted total ROS activity and electrophilicity are presented in Table S5, whereas logP scores (eq 7) for individual compounds are provided in Tables S9–S11.

The relative number of potentially ROS-active molecules was similar in all solvents (Table S5). In the aqueous extract, the major ROS contributors included benzene-1,2,4-triol, 2,3-dihydroxybenzoic acid, and coniferylaldehyde, as well as two HMW aromatics - Figure 7A.

**Figure 7:**
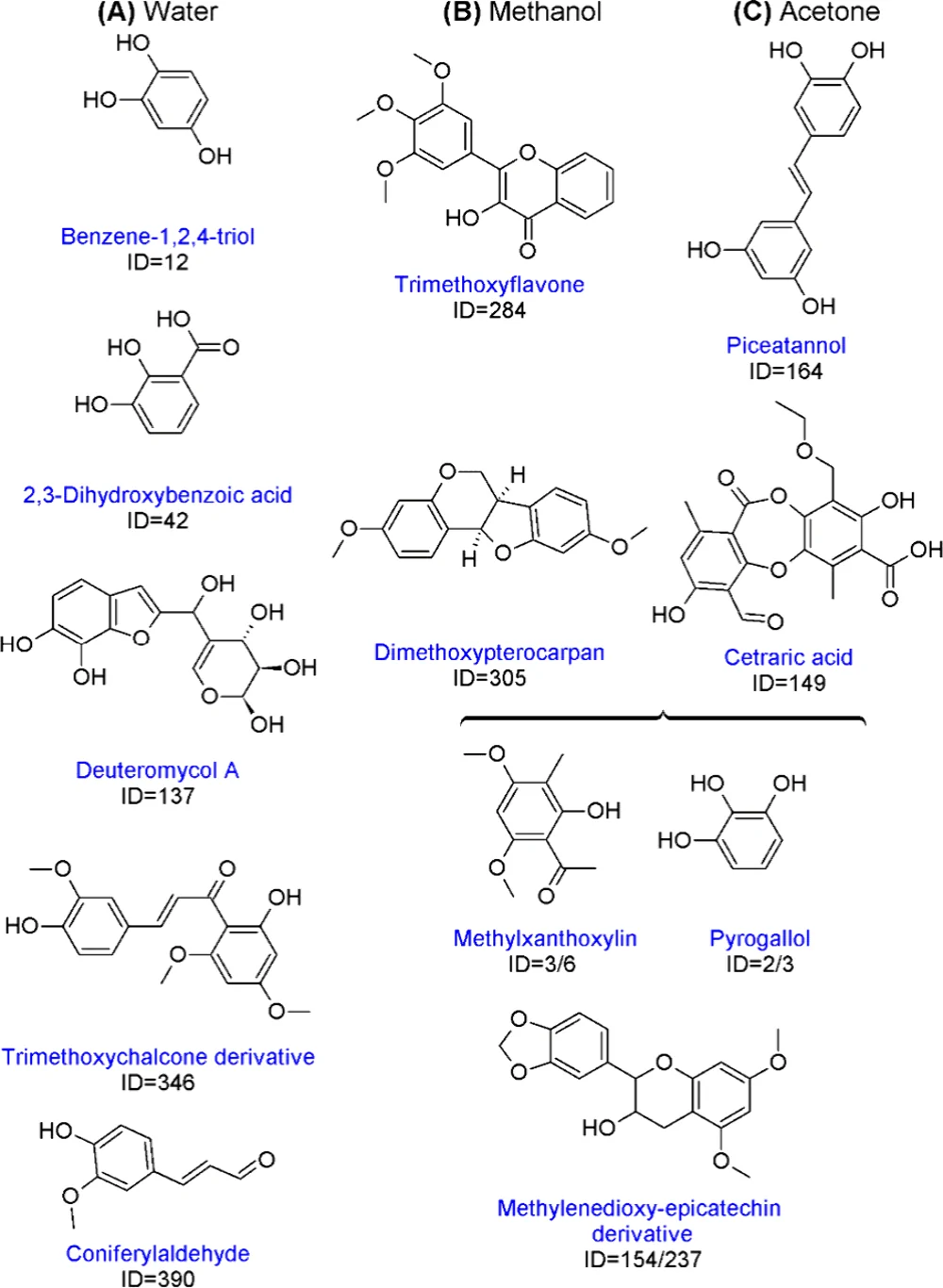
Molecules with the highest ROS contributions (eq 8) tentatively identified in the water (A), methanol (B), and acetone (C) BrC extracts. ID numbers correspond to names in Tables S9–S11.

Likewise, isolated aromatic systems with limited redox cycling efficiencies were more abundant in water-soluble BrC (Figure 7A).^96^ These potential contributors to intracellular OS annotated in the aqueous extract are characterized by low-to-moderate logP values (Tables S4 and S9). Although such molecules can penetrate cells via passive diffusion,^19^ they exhibit lower membrane partitioning, reduced mitochondrial accumulation, and faster efflux.^97^ Therefore, despite a similar proportion of ROS-flagged molecules (Table S5), the effective intracellular OS of the aqueous fraction is likely lower than that of organic extracts.^47^

Conversely, organic extracts, particularly acetone (Figure 7C), were enriched in conjugated aromatic systems. Therefore, the potential contributors to intracellular OS present in methanol and acetone extracts (Figure 7B and C) likely possessed high redox-cycling efficiencies (see also Tables S10 and S11).^96^ The molecules with the highest ROS contributions (eq 8) include methylxanthoxylin, pyrogallol, and a methylenedioxy-epicatechin derivative, which, together with the solvent-specific tracers, all possessed (conjugated) catechol or polyoxygenated aromatic moieties (Figures 7B and C). Such compounds can facilitate redox cycling and stabilization of semiquinone intermediates, amplifying intracellular OS.^98,99^ Furthermore, the higher logP values of ROS-active tracers in organic extracts (Tables S10 and S11) favor partitioning into cellular membranes, enhancing intracellular exposure while also increasing the baseline narcosis.^41,47^ The aqueous extracts exhibited the highest proportion of protein-binding alerts (Table S5). At the same time, the lipophilic electrophiles in organic solvents are more likely to reach higher effective intracellular concentrations, analogous to the potentially ROS-active molecules.^47,97^ The aqueous fraction showed the lowest hydrophobicity, with median logP = 1.48, compared with 2.72 (methanol) and 3.09 (acetone) - Table S4.

The solvent-dependent progression from metabolic inhibition to membrane-compromised cellular injury parallels the increase in extract hydrophobicity in the order of water, methanol, and acetone (Table S4), supporting the proposed contribution of more lipophilic BrC constituents to the higher cytotoxicity of the organic fractions. Hence, compounds with the highest logP scores (Table S12) were further analyzed to identify molecular tracers contributing to the cytotoxicity of BrC – Table 2.

**Table 2:** Combined List of Harmful BrC Tracers with the Highest logP Score Values in All Extracts^a^

	Chemical	Cytotoxicity		
Name^a^	classification	Reported	Evidence^b^ ^,^ ^c^	ref.
3-Hydroxybenzoic acid	Benzoic acids	Low	IC_50_ (A549) > 100 μg mL^–1^	^100^
2,3-Dihydroxybenzoic acid	Benzoic acids	Low	IC_50_ (MCF-7 and MDA-MB-231) = 630 −2050 μg mL^–1^	^101^
Salicylic acid	Benzoic acids	Low	IC_50_ (A549) = 830 μg mL^–1^	^102^
p-Coumaric acid	Cinnamic acids	Moderate	IC_50_ (A549) = 67.6 μg mL^–1^; N2a apoptosis at 25 μg mL^–1^.	^103,104^
Benzene-1,2,4-triol	Quinols	High	Marked apoptosis (HL-60) = 6.31 μg mL^–1^	^105^
2’,4-Dihydroxy-3,4’,6’-trimethoxychalcone	Chalcones	Moderate to high	Related chalcones: IC_50_ (HepG2) = 18.7 μg mL^–1^	^106,107^
6-Methoxy-7-(3-methylbut-2-enoxy)chromen-2-one	Coumarins	Moderate to high	Related coumarins: IC_50_ (MCF-7 and MDA-MB-231) = 31–40 μg mL^–1^	^108^
Columbianetin	Angular furanocoumarins	Moderate to high	IC_50_ (Human dermal fibroblasts) = 23.1 μg mL^–1^	^109^
Methylxanthoxylin	Phenylketones		No data	
Heteropeucenin, methyl ether	Chromones	Moderate to high	Related chromones IC_50_ (A549) = 18–31 μg mL^–1^	^110^
3-Phenyllactic acid	Phenylacetic acids	Low	No cytotoxicity observed up to 2000 μg mL^–1^ (Caco-2).	^111^
Coniferylaldehyde	Methoxyphenols	Undetermined	Nrf2/ARE and proliferation-related effects reported.	^112^
3,9-Dimethoxypterocarpan	Pterocarpans	Moderate	IC_50_ (A549) for related pterocarpans: > 5 μg mL^–1^	^113^
7-Hydroxy-2’,4’,5′-trimethoxyisoflavan	Isoflavans	Low to very low	Related methoxylated flavonoids showed low or no cytotoxicity	^107^
Agelasine	Terpenoid purine alkaloids	Highly potent	IC_50_ (human cancer cells) = 1.5–10 μg mL^–1^	^114,115^
2-[(2R,8S,8aR)-8,8a-dimethyl-2,3,5,6,7,8-hexahydro-1H-naphthalen-2-yl]prop-2-enoic acid	Bicyclic diterpenoid acids		No data	
Idarubicin	Anthracyclines	Highly potent	Potent cytotoxicity	^116^
Isomer of β-sitosterol	Stigmastanes and derivatives	Moderate to high	IC_50_ (A549) for β-sitosterol = 12 μg mL^–1^	^117^
Boldenone undecylenate	Steroid esters	Activity confirmed	Observed oxidative stress and apoptosis	^118^
SB 205607			No data	

a Candidates are listed in the order from highest to lowest logP score (eq 7) – see Table S12.

b Caco-2, human colorectal adenocarcinoma cells; HepG2, human hepatocellular carcinoma cells; HL-60, human promyelocytic leukemia cells; MCF-7 and MDA-MB-231, human breast cancer cells; N2a, mouse neuroblastoma cells; ARE, antioxidant response element; Nrf2, nuclear factor erythroid 2-related factor 2. Values for related compounds or other cell lines are provided only when compound-specific A549 data were unavailable.

c Exposure times varied from 8 to 72 h and are not listed for clarity.

d LogP scores and their relative and cumulative contributions are listed in Table S12.

In Table 2, the main contributors to predicted baseline toxicity included 3-hydroxybenzoic, 2,3-dihydroxybenzoic, salicylic, p-coumaric acids, methoxyphenols, quinols, chalcones and coumarins. Experimental cytotoxicity or related biological activity has been reported for several of these compounds or their structural analogues.^100–108^ While hydroxybenzoic and cinnamic acids exhibit moderate to low toxicity,^100–104^ they were abundant in BrC (Tables S6–S8), resulting in high logP scores. As discussed in section 3.2, these tracers were previously identified in BB emissions, but their presence was not explicitly associated with the toxic properties of fine BrC. Moreover, moderate to high cytotoxic potency has been reported for tracers classified as chromones,^110^ pterocarpans,^113^ terpenoid purine alkaloids,^114,115^ anthracyclines,^116^ stigmastanes,^117^ and some steroid esters,^118^ supporting the use of predicted logP values for candidate prioritization.^47,57^ At the same time, a few tracers with relevant logP scores, phenyllactic acid, 7-hydroxy-2’,4’,5′-trimethoxyisoflavan (isoflavan), exhibited low reported cytotoxicity,^107,111^ or, in the case of methylxanthoxylin (phenylketone), SB 205607 (steroid ester), lacked cytotoxicological characterization. Nevertheless, data presented in Table 2 indicate that plant sterols and structurally related compounds may contribute to the cytotoxicity of BrC.

In Table 2, no single chemical class could be associated with the cytotoxic potency of BrC, as the highest predicted logP (Table S12) and ROS- and protein-binding panel scores (Tables S9–S11) were distributed among different chemical classes – see also Appendix 1.^50^ Such a result is consistent with toxicity frameworks in which nonspecific baseline cytotoxicity is governed largely by partitioning into cellular membranes, whereas excess toxicity depends on additional structural features associated with redox activity, electrophilicity, or specific molecular interactions.^47,49,97^ Hence, compounds from various classes likely contribute to the total cytotoxicity via different mechanisms. The contributors listed in Tables 2 and S12 included lipophilic steroidal, lipid-like, terpenoid and prenylated aromatic molecules, with conjugated phenolic structures having ROS- or protein-binding potential. Therefore, combinations of lipophilicity, abundance and redox-active or electrophilic structural motifs appear more informative for analyzing the distribution of cytotoxic potential in BrC than broad chemical-class assignment alone.^100,107,108^

## 4 Conclusions

This study investigated the molecular composition and toxicity of fine BrC aerosol generated by biomass pyrolysis at 350 °C, close to the wood ignition temperature. Chemical analyses were performed with an LC–HRMS–NTA protocol, whereas cytotoxicity was studied using MTT, LDH, and Annexin V/PI assays.

Between 776 and 1132, molecular features were detected depending on the extraction solvent, among which 611–989 elemental formulas were identified. The molecular structures assigned using NTA were primarily lignin-derived phenols, aromatic acids, and other oxygenated aromatics, consistent with freshly emitted tar-like BrC. Molecular metrics (Van Krevelen space, DBE, Kendrick mass defect, and carbon oxidation state) showed that organic solvents preferentially extracted less oxidized and more hydrophobic compounds, whereas the aqueous fraction was enriched in more polar species.

Complementary in vitro assays showed that BrC cytotoxicity against A549 lung epithelial cells was solvent-dependent and increased in the order water < methanol < acetone, in the concentration range 0–600 μg mL^–1^. This pattern is consistent with the cytotoxic potency of fine BrC estimated for the molecular tracers assigned by NTA. The predicted baseline narcosis showed a higher abundance of lipophilic and conjugated aromatic compounds in the organic extracts. Such molecules are more likely to accumulate in cellular membranes and participate in redox cycling, enhancing OS and baseline narcosis. In contrast, the aqueous fraction contained more polar phenols and aromatic acids with lower membrane affinity and likely shorter intracellular residence times. The potential contributors to baseline narcosis identified in this work included benzoic and cinnamic acids, methoxyphenols, and quinols, but also natural compounds such as flavonoids, coumarins, indoles, and plant sterols.

This work showed that BrC produced by wood pyrolysis close to the ignition temperature (350 °C) may contain a smaller contribution from water-soluble redox-active species compared to PM emitted at higher temperatures. This showcases the importance of source characteristics in constraining the toxicological properties of BB emissions.

At the same time, several limitations of the presented approach should be noted. Molecular annotations obtained by NTA are always tentative, whereas semiquantification with surrogate standards introduces significant uncertainties. Also, the presented work focuses on solvent-extractable organic fractions without considering inorganic components, particularly metal cations. Nevertheless, this work first demonstrates that integrating comprehensive molecular characterization with toxicological screening performed using low-cost computations enables mapping of cytotoxic potential within the chemical space of BB aerosols. The presented results provide new insights into the toxicity of BrC and emphasize the importance of comprehensive toxicological screening of organic pollutants. The data acquired strongly indicate that due to the increasing wildfire activity, many still unrecognized pollutants can adversely affect public health.

## Supplementary Material



## Data Availability

All NTA results for fine BrC extracted with water, methanol, and acetone are freely available in the RepOD open data repository.^50^

## Notes

List of materials and reagents, additional experimental details; mass spectra libraries used, formulas for deriving general properties and chemical classifications based on elemental composition, number of features detected and annotated with NTA, logP and structural-alert data for the detected features, statistical analyses of MTT and LDH results (PDF)
