# Supplementary material for: Fine Brown Carbon Aerosol Emitted by Wood Pyrolysis - New Harmful Tracers and Cytotoxicity in Human Lung Cells
Source: Chem Res Toxicol. 2026 Oct 1;39(10):2063–77. doi: 10.1021/acs.chemrestox.6c00273 (PMC13685490; doi:10.1021/acs.chemrestox.6c00273)
Supplement: Supplementary file 1 [file tx6c00273_si_001.pdf]

Supporting Information

Fine brown carbon aerosol emitted by wood pyrolysis - new harmful tracers and cytotoxicity in human lung cells

Nadira Zendi†, Vinh Nguyen†, Bartłomiej Witkowski†*, Anna Pogorzelska‡, Małgorzata Milczarek‡, Tomasz Gierczak†

†University of Warsaw, Faculty of Chemistry, al. Żwirki i Wigury 101, 02-089 Warsaw, Poland

‡Department of Biomedical Research, National Medicines Institute, 00-725 Warsaw, Poland

Corresponding Author:*bwitk@chem.uw.edu.pl

Contents:

List of materials and reagents, additional experimental details; mass spectra libraries used, formulas for deriving general properties and chemical classifications based on elemental composition, number of features detected and annotated with NTA, logP and structural-alert data for the detected features, statistical analyses of MTT and LDH results. 35 pages, 1 figure, 16 tables.

S1. Materials and reagents

Commercial mixed-wood pellets were obtained from the Polish Coal Group and met the requirements of EN ISO 17225-2, class A1: diameter, 6–8 mm; moisture content, ≤10%; and ash content, ≤0.7%. The pellets were intended for domestic boilers and fireplaces and were stored at room temperature under dry, dark conditions. The producer did not assign a catalogue number to this commercial fuel. A549 (CCL-185) lung carcinoma cell line was obtained from American Type Culture Collection (ATCC, USA). F-12k Nut Mix medium, Dulbecco's Phosphate-buffered saline (DPBS), and trypan blue solution staining were obtained from Gibco Life Technologies, USA. Fetal bovine serum (FBS, S181H-500), 1% antibiotic solution (10,000 U mL^{-1} penicillin, 10 mg mL^{-1} streptomycin, 25 µg mL^{-1} amphotericin B), and non-essential amino acids (10 mM, X0557), were obtained from Biowest, South America. 3-(4,5-dimethylthiazol-2-yl)-2,5-diphenyltetrazolium bromide, and isopropanol were obtained from Sigma-Aldrich. Acetonitrile, methanol, water, and formic acid (98%) were purchased from Sigma–Aldrich, (Germany). Acetone was purchased from Fisher Scientific (Chemat, Poland). Nitrogen (≥99.999%) was supplied by Multax (Stare Babice, Poland). Isatin (99%), succinic (>99%), adipic (99%), and caffeic acid (99%) were purchased from Sigma – Aldrich, Germany; fenuron (99%) and isoproturon (99%) were from LGC, England; isoleucine and tryptophan were research grade from Serva, Germany; tetradecanedioic acid (98%) was purchased from Alfa Aesar, USA; sebacic acid (>95%) was purchased from Honeywell Fluka, USA.

Table S1. Surrogate standards used for semi-quantitative analyses

Name	Retention time (min)	Retention time range covered		Nominal m/z	Ionization mode in ESI
		Start	End		
Isoleucine	2.2	0	5	132	Positive
Tryptophan	10.3	5	15	205	
Isatin	17.5	15	25	148	
Fenuron	20.4	25	35	165	
Isoproturon	38.5	35	50	207	
Succinic acid	2.8	0	5	117	Negative
Adipic acid	9.8	5	15	129	
Caffeic acid	17.9	15	25	179	
Sebacic acid	26.3	25	35	201	
Tetradecanedioic acid	39.3	35	50	257	

S2. Mass spectra libraries used in the non-target analysis workflow

Table S2. MS databases used in the non-target analysis workflow

Name	Reference
Mass Bank of North America	1
MS-DIAL, MSMS_Public_ExpBioInsilico_NEG_VS19	2
MS-DIAL, MSMS_Public_ExpBioInsilico_Pos_VS19	2
Local databases for only MS-FINDER: human metabolites (HMDB, Urine, Saliva, Feces, Serum, CSF, SMPDB), lipids (LipidMAPS), yeast (YMDB), *E.coli* (ECMDB), bovine (BMDB), drug (DrugBank), food (FooDB), plants (PlantCyc), biomolecules (ChEBI, PubChem), toxins (T3DB), environmental compounds (STOFF), blood exposome (BLEXP), and natural products (NPA, NANPDB, COCONUT, KNApSAcK, UNPD).	3

S3. General properties of solvent-extractable brown carbon aerosol derived from the HR-MS data

Double Bond Equivalents (DBE), Carbon oxidation state (OS_c), Kendrick mass, and Kendrick mass defect were derived for features annotated at least at level 4 (elemental formula assignment – see section 2.3.1 in the main text) using Eqs. S1-S4 [4-7].

$$DBE = \frac{1}{2}(2 * n_C - n_H + n_N - n_{halogen} + 2) \quad (S1)$$

$$OS_c = (2 \times n_O + 2 \times n_s - n_H + 3 \times n_N)/n_C \quad (S2)$$

Kendrick mass = IUPAC mass × (14/14.01565) (S3)

Kendrick mass defect = nominal Kendrick mass - exact Kendrick mass (S4)

In Eqs. n_c, n_O, n_S, n_H, n_N, $n_{halogen}$, are the number of carbon, oxygen, sulfur, hydrogen, nitrogen, and halogen atoms in each feature annotated at level 4 or lower – see section 2.3.1 in the main text. IUPAC mass is the atomic or molecular mass calculated using the standard atomic weights.

The annotated features were assigned to six classes: hydrocarbons, carbohydrates, lignins, lipids, peptides, and tannins, based on the H/C and O/C ratios – Table S3 [8, 9].

Table S3. H/C and O/C ratio values for different chemical classes of BrC tracers

Chemical class		Hydrocarbon (condensed)	Carbohydrate	Lignin	Lipid	Peptide	Tannin
H/C	Min.	< 1.1	1.53	0.86	1.34	1.34	0.70
	Max.		2.20	1.34	2.18	2.18	1.01
O/C	Min.	< 0.2	0.56	0.21	0.01	0.17	0.16
	Max.		1.23	0.44	0.35	0.48	0.84

S4. Number of features extracted and identified via non-target analyses in the solvent-extractable fine BrC

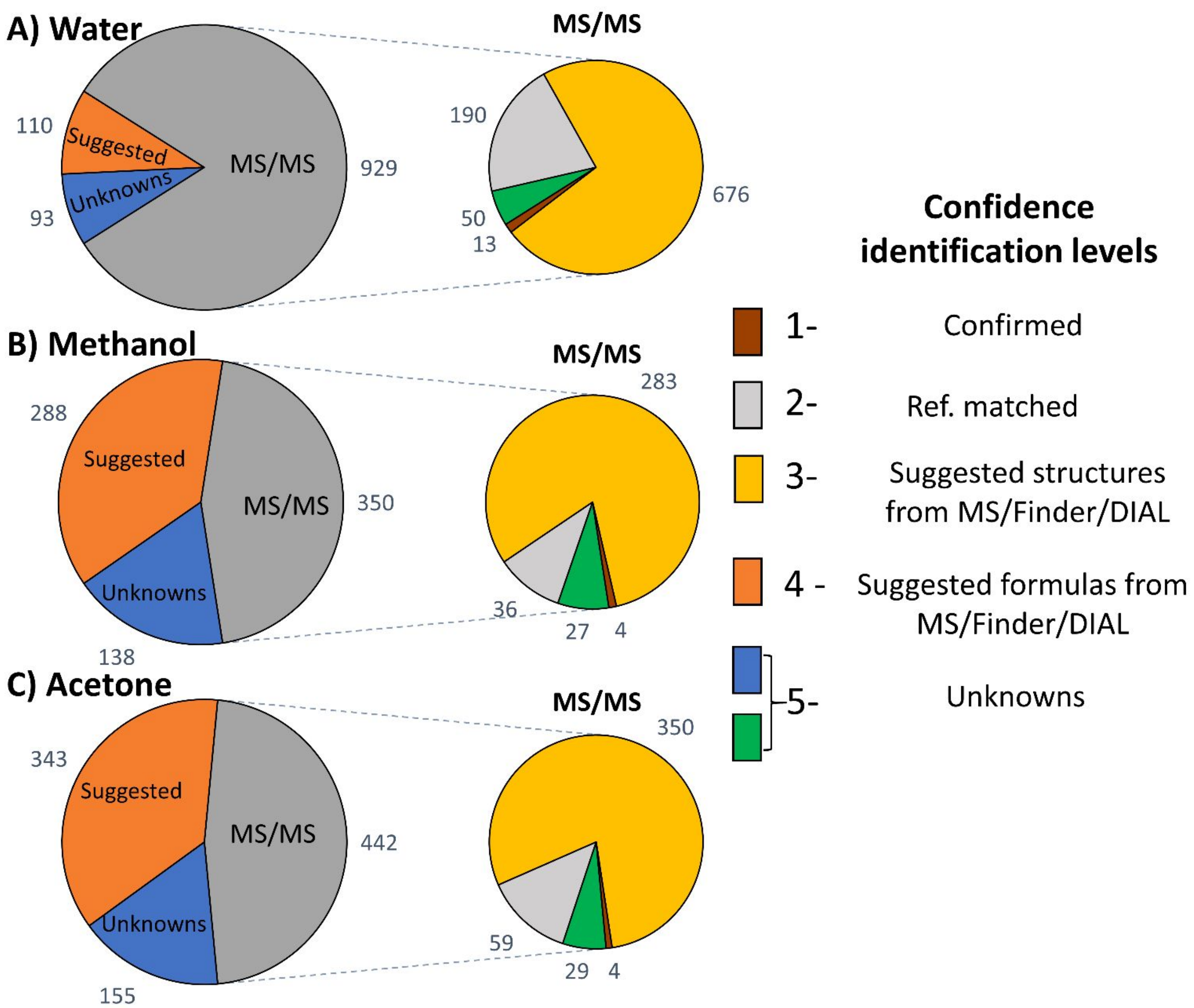

Fig. S1. The number of features extracted and annotated with the NTA workflow in the aqueous (A), methanol (B), and acetone (C) extracts.

Table S4. Predicted logP-values for the cumulative 80% most abundant molecules in each extract

Scales	Ranges	Solvent (number of 80% most abundant molecules in the extract)		
		Water (98)	Methanol (62)	Acetone (64)
LogP	*LogP ranges*	*LogP relative contribution*		
Very hydrophilic	<0	13.3%	3.2%	4.7%
Hydrophilic	0-1	21.4%	12.9%	4.7%
Moderately lipophilic	1-3	42.9%	37.1%	34.4%
Lipophilic	3-5	20.4%	40.3%	43.8%
Very lipophilic	>5	2.0%	6.5%	12.5%
Median values		1.48	2.72	3.09

Table S5. Number of flagged compounds in the three solvent extracts in the ROS and protein binding panels

Solvent	Predicted ROS activity	
	Number of flagged molecules	*Relative contribution of flagged molecules*
Water	30	45%
Methanol	26	46%
Acetone	28	50%
	**Predicted protein binding potential**	
Water	33	18%
Methanol	18	13%
Acetone	18	14%

S5. Molecules tentatively identified in the filter extract corresponding to the 80% cumulative concentration of organic compounds detected with LC-ESI/MS

Table S6. Tentatively identified tracers of fine BrC in the aqueous extract

Mass (mg)	Name	SMILES	Classification
0.172	3-hydroxybenzoic acid	C1=CC(=CC(=C1)O)C(=O)O	tannin
0.105	Benzene-1,2,4-triol	OC1=CC=C(O)C(O)=C1	tannin

Mass (mg)	Name	SMILES	Classification
0.073	Idarubicin	C1=CC=C2C(=C1)C(=C(N2)O)C3=NC4=CC=CC=C4C3=O	soot
0.067	p-Coumaric acid	C1=CC(=CC=C1/C=C/C(=O)O)O	lignin
0.048	2,3-Dihydroxybenzoic acid	C1=CC(=C(C(=C1)O)O)C(=O)O	tannin
0.038	3-PHENYLLACTIC ACID	C1=CC=C(C=C1)CC(C(=O)O)O	lignin
0.035	Deuteromycol A	C1=CC(=C(C2=C1C=C(O2)C(C3=CO[C@@H]([C@@H]([C@H]3O)O)O)O)O)O	tannin
0.033	UNPD123033	C(C=O)C(C(CO)O)O	tannin
0.031	Coniferylaldehyde	COC1=CC(\C=C\C=O)=CC=C1O	lignin
0.030	cis-4-Carboxymethylenebut-2-en-4-olide	O=C(O)C=C1OC(=O)C=C1	0
0.028	Thymine	CC1=CNC(=O)NC1=O	lignin
0.026	Methylxanthoxylin	O=C(C=1C(O)=C(C(OC)=CC1OC)C)C	lignin
0.026	4-Thiouridine	C1=CN(C(=O)NC1=S)[C@H]2[C@@H]([C@@H]([C@H](O2)CO)O)O	0
0.025	Vanillomandelic acid	COC1=C(C=CC(=C1)C(C(=O)O)O)O	0
0.025	4-Hydroxy-3-methoxycinnamaldehyde	COC1=C(C=CC(=C1)/C=C/C=O)O	lignin
0.024	1,3 Cyclohexanedione	O=C1CC(=O)CCC1	lignin
0.022	Zonisamide	C1=CC=C2C(=C1)C(=NO2)CS(=O)(=O)N	lignin
0.022	MANNITOL	OC[C@@H](O)[C@@H](O)[C@H](O)[C@H](O)CO	0
0.020	Alibendol	C1=C(C(=C(C=C1CC=C)C(=O)NCCO)O)OC	lignin
0.020	Azelaic acid (Not validated)	C(CCCC(=O)O)CCCC(=O)O	peptide
0.019	Heteropeucenin, Methyl Ether	CC1=CC(=O)C2=C(O1)C(=C(C=C2O)OC)CC=C(C)C	lignin
0.017	Mandelic acid	C1=CC=C(C=C1)C(C(=O)O)O	lignin
0.017	Glutaric acid	OC(=O)CCCC(O)=O	carbohydrate
0.016	Acetovanillone;Apocynin	O=C(O)C=CC(=CC(=O)O)C(O)CC(=O)O	lignin
0.016	2',4-Dihydroxy-3,4',6'-Trimethoxychalcone	COC1=CC(=C(C(=C1)OC)C(=O)/C=C/C2=CC(=C(C=C2)O)OC)O	lignin
0.015	4-hydroxycoumarin	O=C1OC=2C=CC=CC2C(O)=C1	0
0.015	Sinapoyl aldehyde	COC1=CC(\C=C\C=O)=CC(OC)=C1O	lignin
0.015	Methylxanthoxylin	O=C(C=1C(O)=C(C(OC)=CC1OC)C)C	lignin
0.014	Cumate	CC(C)C1=CC=C(C=C1)C(=O)[O-]	0

Mass (mg)	Name	SMILES	Classification
0.014	columbianetin	CC(C)([C@@H]1CC2=C(O1)C=CC3=C2OC(=O)C=C3)O	lignin
0.013	Syringaldehyde	O=Cc(c1)cc(OC)c(O)c(OC)1	0
0.013	Catechol	C1=CC=C(C(=C1)O)O	lignin
0.013	6-methoxy-7-(3-methylbut-2-enoxy)chromen-2-one	CC(=CCOC1=C(C=C2C=CC(=O)OC2=C1)OC)C	lignin
0.013	2-(1H-pyrrol-1-ylcarbonyl)benzene-1,3,5-triol	C1=CN(C=C1)C(=O)C2=C(C=C(C=C2O)O)O	tannin
0.012	Acetosyringone	O=C(C1=CC(OC)=C(O)C(OC)=C1)C	lignin
0.011	ethyl 2,3-dioxo-1,4-dihydroquinoline-4-carboxylate	O=C(OCC)C1C(=O)C(=O)NC=2C=CC=CC21	lignin
0.011	vanillic acid	O=C(O)C1=CC=C(O)C(OC)=C1	tannin
0.011	Chavicol O-beta-glucopyranoside	C=CCC1=CC=C(C=C1)OC2C(C(C(C(O2)CO)O)O)O	lignin
0.010	Succinic acid	OC(=O)CCC(O)=O	0
0.010	Vanillin	O=CC1=CC=C(O)C(OC)=C1	lignin
0.009	Orcinol	CC1=CC(=CC(=C1)O)O	lignin
0.009	2-[(2R,8S,8aR)-8,8a-dimethyl-2,3,5,6,7,8-hexahydro-1H-naphthalen-2-yl]prop-2-enoic acid	C[C@H]1CCCC2=CC[C@H](C[C@]12C)C(=C)C(=O)O	lipid
0.009	6-Methoxy-7-hydroxycoumarin	COC1=C(C=C2C(=C1)C=CC(=O)O2)O	tannin
0.009	1-(2,4-dihydroxyphenyl)-2-(3,5-dimethoxyphenyl)propan-1-one	CC(C1=CC(=CC(=C1)OC)OC)C(=O)C2=C(C=C(C=C2)O)O	lignin
0.008	Dehydrosalsolidine	CC1=NCCC2=CC(=C(C=C12)OC)OC	0
0.008	7-Hydroxy-2',4',5'-trimethoxyisoflavan	COC1=CC(=C(C=C1C2CC3=C(C=C(C=C3)O)OC2)OC)OC	lignin
0.008	Tetramisole	C1CSC2=NC(CN21)C3=CC=CC=C3	0
0.008	UNPD110798	O=C1OC(C2=COC(=CC2=O)CC(O)C)C=3C(OC)=CC(OC)=CC13	lignin
0.008	6-Nitrobenzimidazole	C1=CC2=C(C=C1[N+](=O)[O-])NC=N2	tannin
0.007	no MS2: gamma-sitosterol	CC[C@@H](CC[C@@H](C)[C@H]1CC[C@@H]2[C@@]1(CC[C@H]3[C@H]2CC=C4[C@@]3(CC[C@@H](C4)O)C)C)C(C)C	lipid
0.007	Sinapoyl aldehyde	COC1=CC(\C=C\C=O)=CC(OC)=C1O	lignin
0.007	Cumyluron	CC(C)(C1=CC=CC=C1)NC(=O)NCC2=CC=CC=C2Cl	0
0.007	Umbelliferone	C1=CC(=CC2=C1C=CC(=O)O2)O	0
0.007	Undecanedioic acid	C(CCCCC(=O)O)CCCCC(=O)O	peptide

Mass (mg)	Name	SMILES	Classification
0.007	Kanakugiol	COC1=C(C(=C(C(=C1C(=O)/C=C/C2=CC=CC=C2)O)OC)OC)OC	lignin
0.007	Phloretin	C1=CC(=CC=C1CCC(=O)C2=C(C=C(C=C2O)O)O)O	lignin
0.007	5-O-Methylembelin	CCCCCCCCCCCC1=C(C(=O)C=C(C1=O)OC)O	lipid
0.007	Boldenone Undecylenate	CC12CC[C@H]3C([C@@H]1CC[C@@H]2OC(=O)CCCCCCCCC=C)CCC4=CC(=O)C=CC34C	lipid
0.006	Syringaldehyde	O=Cc(c1)cc(OC)c(O)c(OC)1	
0.006	Agelasine (Stereochemistry Of Diterpene Unknown)	CC1CCC2([C@H]([C@]1(C)CC/C(=C/C[N+]3=CN(C4=C(N=CN=C43)N)C)/C)CCC=C2C)C.[Cl-]	
0.006	SB 205607	CN1CC[C@@]2(CC3=NC4=CC=CC=C4C=C3C[C@H]2C1)C5=CC(=CC=C5)O.O.Br	
0.006	2-(2-hydroxypropan-2-yl)-6-(2-methylbut-3-en-2-yl)-2H,3H,7H-furo[3,2-g]chromen-7-one	CC(C)(C=C)C1=CC2=CC3=C(C=C2OC1=O)O[C@@H](C3)C(C)(C)O	lipid
0.006	2-methoxy-1,4-naphthoquinone	COC1=CC(=O)C2=CC=CC=C2C1=O	tannin
0.006	Corynoline	C[C@]12[C@H](CC3=CC4=C(C=C3[C@H]1N(CC5=C2C=CC6=C5OCO6)C)OCO4)O	lignin
0.006	methyl 1-methyl-9H-beta-carbolin-7-yl ether	N=1C=CC=2C=3C=CC(OC)=CC3NC2C1C	soot
0.006	alpha,beta-Dihydroresveratrol	C1=CC(=CC=C1CCC2=CC(=CC(=C2)O)O)O	lignin
0.006	(1R,9S,10S)-3,4-dihydroxy-11,11-dimethyl-5-(propan-2-yl)-16-oxatetracyclo[7.5.2.0?,??.0?,?]hexadeca-2(7),3,5-triene-8,15-dione	CC(C)C1=C(C(=C2C(=C1)C(=O)[C@@H]3[C@@H]4[C@@]2(CCCC4(C)C)C(=O)O3)O)O	lignin
0.006	methyl 2-ethyl-4-[(3R,4R,5S)-5-hydroxy-4,5-dimethyl-2-oxooxolan-3-yl]-2-methyl-3-oxobutanoate	CC[C@@](C)(C(=O)C[C@@H]1[C@H]([C@@](OC1=O)(C)O)C)C(=O)OC	peptide
0.006	NCGC00169706-02!6-ethyl-2,4-dihydroxy-3-methylbenzaldehyde	CCC1=C(C=O)C(O)=C(C)C(O)=C1	lignin
0.006	Triethylphosphate	CCOP(=O)(OCC)OCC	
0.006	8-Methylsulfinyloctyl isothiocyanate	CS(=O)CCCCCCCCN=C=S	lipid
0.006	Levetiracetam	CC[C@@H](C(=O)N)N1CCCC1=O	lipid
0.006	Tomelukast	CCCC1=C(C=CC(=C1O)C(=O)C)OCCCCC2=NNN=N2	lipid
0.006	Euparin	CC(=C)C1=CC2=CC(=C(C=C2O1)O)C(=O)C	lignin
0.006	4-methoxy-9-(2-methylbut-3-en-2-yl)furo[3,2-g]chromen-7-one	CC(C)(C=C)C1=C2C=CC(=O)OC2=C(C3=C1C=CO3)OC	lignin

Mass (mg)	Name	SMILES	Classification
0.006	Matairesinol	COC1=C(C=CC(=C1)C[C@H]2COC(=O)[C@@H]2CC3=CC(=C(C=C3)O)OC)O	lignin
0.005	aurapten	CC(=CCC/C(=C/COC1=CC2=C(C=C1)C=CC(=O)O2)/C)C	
0.005	Caffeic acid	C1=CC(=C(C=C1/C=C/C(=O)O)O)O	tannin
0.005	Isosakuranin	COC1=CC=C(C=C1)[C@@H]2CC(=O)C3=C(C=C(C=C3O2)O[C@H]4[C@@H]([C@H]([C@@H]([C@H](O4)CO)O)O)O)O	
0.005	PYROGLUTAMATE	OC(=O)[C@@H]1CCC(=O)N1	
0.005	Virensic acid	CC1=CC(=C(C2=C1C(=O)OC3=C(O2)C(=C(C(=C3C)O)C(=O)O)C)C=O)O	tannin
0.005	Angelic anhydride	O=C(OC(=O)C(=CC)C)C(=CC)C	lipid
0.005	SUBERIC ACID	O=C(O)CCCCCCC(=O)O	
0.005	4-Methyldaphnetin	CC1=CC(=O)OC2=C1C=CC(=C2O)O	tannin
0.005	Ethyl vanillin	[H]C(=O)c1ccc(O)c(OCC)c1	lignin
0.005	Ethylparaben	O=C(OCC)c1ccc(O)cc1	lignin
0.005	2,3-Dihydroxybiphenyl	C1=CC=C(C=C1)C2=C(C(=CC=C2)O)O	tannin
0.005	Cer 11:1;2O/2:0	CCCCCC/C=C/C(C(CO)NC(=O)C)O	lipid
0.005	ARABITOL	C([C@H](C([C@@H](CO)O)O)O)O	
0.005	Protocatechuic acid	O=C(O)c1ccc(O)c(O)c1	tannin
0.005	1-(2-hydroxy-4,6-dimethoxyphenyl)ethanone	O=C(c1c(O)cc(OC)cc1(OC))C	lignin
0.005	3-(3,4-Dimethoxyphenyl)-2-propenoic acid	O=C(O)C=CC1=CC=C(OC)C(OC)=C1	lignin
0.004	Phthalic anhydride	C1=CC=C2C(=C1)C(=O)OC2=O	
0.004	5,7-Dimethoxy-4-methylcoumarin	CC1=CC(=O)OC2=C1C(=CC(=C2)OC)OC	lignin
0.004	Chlorogenoquinone (Not validated)	C1C(C(C(CC1(C(=O)O)O)OC(=O)/C=C\C2=CC(=O)C(=O)C=C2)O)O	tannin
0.004	Benzoic acid	C1=CC=C(C=C1)C(=O)O	tannin
0.004	ERYTHRITOL	C([C@H]([C@H](CO)O)O)O	
0.004	Gaultherin	O=C(OC)c3ccccc3(OC2OC(COC1OCC(O)C(O)C1(O))C(O)C(O)C2(O))	

Table S7. Tentatively identified tracers of fine BrC in the methanol extract

Mass (mg)	Name	SMILES	Classification
0.833	Methylxanthoxylin	O=C(C=1C(O)=C(C(OC)=CC1OC)C)C	lignin

Mass (mg)	Name	SMILES	Classification
0.479	Salicylic acid	C1=CC=C(C(=C1)C(=O)O)O	tannin
0.398	Boldenone_Undecylenate	CC12CC[C@H]3C([C@@H]1CC[C@@H]2OC(=O)CCCCCCCCC=C)CCC4=CC(=O)C=CC34C	lipid
0.330	Triethylphosphate	CCOP(=O)(OCC)OCC	
0.311	Pyrogallol	C1=CC(=C(C(=C1)O)O)O	tannin
0.262	3,9-Dimethoxypterocarpan	COC1=CC2=C(C=C1)[C@@H]3COC4=C([C@@H]3O2)C=CC(=C4)OC	lignin
0.244	SB 205607	CN1CC[C@@]2(CC3=NC4=CC=CC=C4C=C3C[C@H]2C1)C5=CC(=CC=C5)O.O.Br	
0.236	p-Coumaric acid	C1=CC(=CC=C1/C=C/C(=O)O)O	lignin
0.200	2-[(2R,8S,8aR)-8,8a-dimethyl-2,3,5,6,7,8-hexahydro-1H-naphthalen-2-yl]prop-2-enoic acid	C[C@H]1CCCC2=CC[C@H](C[C@]12C)C(=C)C(=O)O	lipid
0.188	Heteropeucenin, Methyl Ether	O=C1C=C(OC=2C1=C(O)C=C(OC)C2CC=C(C)C)C	lignin
0.182	Enterolactone	C1[C@H]([C@@H](C(=O)O1)CC2=CC(=CC=C2)O)CC3=CC(=CC=C3)O	lignin
0.171	columbianetin	O=C1OC2=C(C=C1)C=CC=3OC(CC32)C(O)(C)C	lignin
0.164	6-methoxy-7-(3-methylbut-2-enoxy)chromen-2-one	O=C1OC=2C=C(OCC=C(C)C)C(OC)=CC2C=C1	lignin
0.162	isoimperatorin	CC(=CCOC1=C2C=CC(=O)OC2=CC3=C1C=CO3)C	lignin
0.152	(??)-3',4'-Methylenedioxy-5,7-dimethylepicatechin	OC1CC=2C(OC)=CC(OC)=CC2OC1C3=CC=C4OCOC4=C3	lignin
0.145	NCGC00187465-02!(E)-1-(5,7-dihydroxy-2,2,6-trimethylchromen-8-yl)-3-phenylprop-2-en-1-one	CC1=C(C2=C(C(=C1O)C(=O)/C=C/C3=CC=CC=C3)OC(C=C2)(C)C)O	tannin
0.143	2,8-Phenazinediamine	C1=CC2=C(C=C1N)N=C3C=C(C=CC3=N2)N	soot
0.137	1-(2,4-dihydroxyphenyl)-2-(3,5-dimethoxyphenyl)propan-1-one	CC(C1=CC(=CC(=C1)OC)OC)C(=O)C2=C(C=C(C=C2)O)O	lignin
0.137	m-Xylene-4-sulfonic acid	CC1=CC(=C(C=C1)S(=O)(=O)O)C	lignin
0.137	aurapten	CC(=CCC/C(=C/COC1=CC2=C(C=C1)C=CC(=O)O2)/C)C	
0.136	adrenosterone	C[C@]12CCC(=O)C=C1CC[C@@H]3[C@@H]2C(=O)C[C@]4([C@H]3CCC4=O)C	
0.131	Kaempferol	C1=CC(=CC=C1C2=C(C(=O)C3=C(C=C(C=C3O2)O)O)O)O	

Mass (mg)	Name	SMILES	Classification
0.131	2-(2-hydroxypropan-2-yl)-6-(2-methylbut-3-en-2-yl)-2H,3H,7H-furo[3,2-g]chromen-7-one	CC(C)(C=C)C1=CC2=CC3=C(C=C2OC1=O)O[C@@H](C3)C(C)(C)O	lignin
0.130	Tomelukast	CCCC1=C(C=CC(=C1O)C(=O)C)OCCCCC2=NNN=N2	lipid
0.129	angoletin	CC1=C(C(=C(C(=C1O)C(=O)CCC2=CC=CC=C2)OC)C)O	lignin
0.129	(1R,9S,10S)-3,4-dihydroxy-11,11-dimethyl-5-(propan-2-yl)-16-oxatetracyclo[7.5.2.0?,??.0?,?]hexadeca-2(7),3,5-triene-8,15-dione		lignin
0.128	UNPD110798	O=C1OC(C2=COC(=CC2=O)CC(O)C)C=3C(OC)=CC(OC)=CC13	lignin
0.127	3-Hydroxy-3',4',5'-trimethoxyflavone	COC1=CC(=CC(=C1OC)OC)C2=C(C(=O)C3=CC=CC=C3O2)O	lignin
0.126	cis-4-Carboxymethylenebut-2-en-4-olide	O=C(O)C=C1OC(=O)C=C1	
0.125	(1,9,9b-trihydroxy-6,6,9a-trimethyl-3,5,5a,7,8,9-hexahydro-1H-benzo[e][2]benzofuran-5-yl) hexanoate	CCCCCC(=O)OC1C=C2COC(C2(C3(C1C(CCC3O)(C)C)C)O)O	lipid
0.125	1-(4-methoxyphenyl)isoquinoline	COC1=CC=C(C=C1)C2=NC=CC3=CC=CC=C32	soot
0.124	Octadecanedioic acid	C(CCCCCCCCC(=O)O)CCCCCCCC(=O)O	lipid
0.124	Agelasine (Stereochemistry Of Diterpene Unknown)	CC1CCC2([C@H]([C@]1(C)CC/C(=C/C[N+]3=CN(C4=C(N=CN=C43)N)C)/C)CCC=C2C)C.[Cl-]	
0.123	1,7-bis(3,4-dihydroxyphenyl)-6-hydroxyheptan-3-one	C1=CC(=C(C=C1CCC(=O)CCC(CC2=CC(=C(C=C2)O)O)O)O)O	lignin
0.123	(2S)-Liquiritigenin	C1[C@H](OC2=C(C1=O)C=CC(=C2)O)C3=CC=C(C=C3)O	tannin
0.123	2-methoxy-5-methyl-3-(2-methylbut-3-en-2-yl)chromen-4-one	CC1=C2C(=CC=C1)OC(=C(C2=O)C(C)(C)C=C)OC	
0.123	Phloretin	C1=CC(=CC=C1CCC(=O)C2=C(C=C(C=C2O)O)O)O	lignin
0.122	Dicyclohexyl phthalate	C1CCC(CC1)OC(=O)C2=CC=CC=C2C(=O)OC3CCCCC3	
0.121	2-Hydroxyanthraquinone	C1=CC=C2C(=C1)C(=O)C3=C(C2=O)C=C(C=C3)O	
0.121	Xanthone	C1=CC=C2C(=C1)C(=O)C3=CC=CC=C3O2	soot
0.121	Glutamyl-S-(C3H5)-Cysteine sulfoxide	C/C=C/S(=O)CC(C(=O)O)NC(=O)CCC(C(=O)O)N	
0.121	Isosakuranin	COC1=CC=C(C=C1)[C@@H]2CC(=O)C3=C(C=C(C=C3O2)O[C@H]4[C@@H]([C@H]([C@@H]([C@H](O4)CO)O)O)O)O	

Mass (mg)	Name	SMILES	Classification
0.120	Virensic acid	CC1=CC(=C(C2=C1C(=O)OC3=C(O2)C(=C(C(=C3C)O)C(=O)O)C)C=O)O	tannin
0.120	MGMG 16:3	CC/C=C/C/C=C/C/C=C/CCCCCC(=O)OC(CO)COC1C(C(C(C(O1)CO)O)O)O	peptide
0.120	Coniferaldehyde	COC1=CC(\C=C\C=O)=CC=C1O	lignin
0.120	2,4-Dihydroxybenzophenone	C1=CC=C(C=C1)C(=O)C2=C(C=C(C=C2)O)O	tannin
0.119	Naproxen	C[C@@H](C1=CC2=C(C=C1)C=C(C=C2)OC)C(=O)O	lignin
0.119	ANGOLENSIN (R)	C[C@H](C1=CC=C(C=C1)OC)C(=O)C2=C(C=C(C=C2)O)O	lignin
0.119	agnuside	C1=CO[C@H]([C@H]2[C@@H]1[C@@H](C=C2COC(=O)C3=CC=C(C=C3)O)O)O[C@H]4[C@@H]([C@H]([C@@H]([C@H](O4)CO)O)O)O	
0.118	FA 18:4+2O	O=C(O)CCCCCCCC(=O)C=CC=CC=CC(O)CC	lipid
0.118	Cetraric acid	CCOCC1=C(C(=C(C2=C1OC(=O)C3=C(O2)C(=C(C=C3C)O)C=O)C)C(=O)O)O	tannin
0.117	NCGC00169633-02!9-phenyl-1-(2,4,6-trihydroxyphenyl)nonan-1-one	C1=CC=C(C=C1)CCCCCCCCC(=O)C2=C(C=C(C=C2O)O)O	
0.116	Methyl 1-naphthaleneacetate	COC(=O)CC1=CC=CC2=CC=CC=C21	soot
0.116	5-[6-(3-hydroxy-4-methoxyphenyl)-1,3,3a,4,6,6a-hexahydrofuro[3,4-c]furan-3-yl]-2-methoxyphenol	COC1=C(C=C(C=C1)C2C3COC(C3CO2)C4=CC(=C(C=C4)OC)O)O	lignin
0.116	Biochanin A	COC1=CC=C(C=C1)C2=COC3=CC(=CC(=C3C2=O)O)O	tannin
0.116	Piceatannol	C1=CC(=C(C=C1/C=C/C2=CC(=CC(=C2)O)O)O)O	tannin
0.115	Undecanedioic acid	C(CCCCC(=O)O)CCCCC(=O)O	peptide
0.115	3-(8-hydroxyoctyl)phenol	C1=CC(=CC(=C1)O)CCCCCCCCO	lipid
0.115	2,3-Dihydroxybiphenyl	C1=CC=C(C=C1)C2=C(C(=CC=C2)O)O	tannin
0.114	FA 19:2	CCCCCCCCC/C=C/C=C/CCCCCC(=O)O	lipid
0.112	Cer 8:1;2O/5:0	CCCCC(=O)NC(CO)C(/C=C/CCC)O	lipid
0.111	N-acetyltryptophan	CC(=O)NC(CC1=CNC2=CC=CC=C21)C(=O)O	lignin

Table S8. Tentatively identified tracers of fine BrC in the acetone extract

Mass (mg)	Name	SMILES	Classification
0.677	Methylxanthoxylin	O=C(C=1C(O)=C(C(OC)=CC1OC)C)C	lignin

Mass (mg)	Name	SMILES	Classification
0.348	Boldenone_Undecylenate	CC12CC[C@H]3C([C@@H]1CC[C@@H]2OC(=O)CCCCCCCCC=C)CCC4=CC(=O)C=CC34C	lipid
0.322	Triethylphosphate	CCOP(=O)(OCC)OCC	
0.305	Salicylic acid	C1=CC=C(C(=C1)C(=O)O)O	tannin
0.242	3,9-Dimethoxypterocarpan	COC1=CC2=C(C=C1)[C@@H]3COC4=C([C@@H]3O2)C=CC(=C4)OC	lignin
0.234	11-Dehydrocorticosterone	C[C@]12CCC(=O)C=C1CC[C@@H]3[C@@H]2C(=O)C[C@]4([C@H]3CC[C@@H]4C(=O)CO)C	
0.229	2-[(2R,8S,8aR)-8,8a-dimethyl-2,3,5,6,7,8-hexahydro-1H-naphthalen-2-yl]prop-2-enoic acid	C[C@H]1CCCC2=CC[C@H](C[C@]12C)C(=C)C(=O)O	lipid
0.216	gamma-sitosterol	CC[C@@H](CC[C@@H](C)[C@H]1CC[C@@H]2[C@@]1(CC[C@H]3[C@H]2CC=C4[C@@]3(CC[C@@H](C4)O)C)C)C(C)C	lipid
0.215	Acetovanillone;Apocynin	O=C(O)C=CC(=CC(=O)O)C(O)CC(=O)O	lignin
0.181	Pyrogallol	C1=CC(=C(C(=C1)O)O)O	tannin
0.178	Enterolactone	C1[C@H]([C@@H](C(=O)O1)CC2=CC(=CC=C2)O)CC3=CC(=CC=C3)O	lignin
0.173	Heteropeucenin, Methyl Ether	O=C1C=C(OC=2C1=C(O)C=C(OC)C2CC=C(C)C)C	lignin
0.171	p-Coumaric acid	C1=CC(=CC=C1/C=C/C(=O)O)O	lignin
0.161	columbianetin	O=C1OC2=C(C=C1)C=CC=3OC(CC32)C(O)(C)C	lignin
0.157	6-methoxy-7-(3-methylbut-2-enoxy)chromen-2-one	O=C1OC=2C=C(OCC=C(C)C)C(OC)=CC2C=C1	lignin
0.155	isoimperatorin	CC(=CCOC1=C2C=CC(=O)OC2=CC3=C1C=CO3)C	lignin
0.138	(??)-3',4'-Methylenedioxy-5,7-dimethylepicatechin	COC1=CC2=C(CC(C(O2)C3=CC4=C(C=C3)OCO4)O)C(=C1)OC	lignin
0.136	aurapten	CC(=CCC/C(=C/COC1=CC2=C(C=C1)C=CC(=O)O2)/C)C	
0.135	NCGC00187465-02!(E)-1-(5,7-dihydroxy-2,2,6-trimethylchromen-8-yl)-3-phenylprop-2-en-1-one	CC1=C(C2=C(C(=C1O)C(=O)/C=C/C3=CC=CC=C3)OC(C=C2)(C)C)O	tannin
0.135	2-Methoxyestrone	C[C@]12CC[C@H]3[C@H]([C@@H]1CCC2=O)CCC4=CC(=C(C=C34)OC)O	
0.131	1-(2,6-dihydroxy-4-methoxyphenyl)-3-(4-methoxyphenyl)propan-1-one	COC1=CC=C(C=C1)CCC(=O)C2=C(C=C(C=C2O)OC)O	lignin
0.131	4-Hydroxy-2',4',6'-trimethoxychalcone	COC1=CC(=C(C(=C1)OC)C(=O)C=CC2=CC=C(C=C2)O)OC	lignin

Mass (mg)	Name	SMILES	Classification
0.129	2-(2-hydroxypropan-2-yl)-6-(2-methylbut-3-en-2-yl)-2H,3H,7H-furo[3,2-g]chromen-7-one	CC(C)(C=C)C1=CC2=CC3=C(C=C2OC1=O)O[C@@H](C3)C(C)(C)O	lignin
0.126	angoletin	CC1=C(C(C(=C(C(=C1O)C(=O)CCC2=CC=CC=C2)OC)C)O	lignin
0.125	4-HYDROXYBENZOATE	C1=CC(=CC=C1C(=O)O)O	tannin
0.125	Kaempferol	C1=CC(=CC=C1C2=C(C(=O)C3=C(C=C(C=C3O2)O)O)O)O	
0.125	Malabaricano	CC1C(C(OC1C2=CC(=C(C=C2)O)OC)C3=CC(=C(C=C3)O)OC)C	lignin
0.123	ANGOLENSIN (R)	C[C@H](C1=CC=C(C=C1)OC)C(=O)C2=C(C=C(C=C2)O)O	lignin
0.123	Octadecanedioic acid	C(CCCCCCCCC(=O)O)CCCCCCCC(=O)O	lipid
0.123	(1,9,9b-trihydroxy-6,6,9a-trimethyl-3,5,5a,7,8,9-hexahydro-1H-benzo[e][2]benzofuran-5-yl) hexanoate	CCCCCC(=O)OC1C=C2COC(C2(C3(C1C(CCC3O)(C)C)C)O)O	lipid
0.122	NCGC00385014-01!(E)-1,7-bis(4-hydroxyphenyl)hept-1-en-3-one	C1=CC(=CC=C1CCCCC(=O)/C=C/C2=CC=C(C=C2)O)O	
0.121	Kanakugiol	O=C(C=CC=1C=CC=CC1)C2=C(O)C(OC)=C(OC)C(OC)=C2OC	lignin
0.121	Dicyclohexyl phthalate	C1CCC(CC1)OC(=O)C2=CC=CC=C2C(=O)OC3CCCCC3	
0.121	Phloretin	C1=CC(=CC=C1CCC(=O)C2=C(C=C(C=C2O)O)O)O	lignin
0.120	1,9-dimethyluric acid	CN1C2=C(C(=O)N(C(=O)N2)C)NC1=O	lignin
0.120	Isoflavone base + 3O, 1Prenyl	CC(=CCC1=C(C2=C(C=C1O)OC=C(C2=O)C3=CC=C(C=C3)O)O)C	lignin
0.120	(2S)-Liquiritigenin	C1[C@H](OC2=C(C1=O)C=CC(=C2)O)C3=CC=C(C=C3)O	tannin
0.120	Decursin	CC(=CC(=O)O[C@H]1CC2=C(C=C3C(=C2)C=CC(=O)O3)OC1(C)C)C	lignin
0.120	2-Hydroxyanthraquinone	C1=CC=C2C(=C1)C(=O)C3=C(C2=O)C=C(C=C3)O	
0.119	3-Hydroxy-3',4',5'-trimethoxyflavone	COC1=CC(=CC(=C1OC)OC)C2=C(C(=O)C3=CC=CC=C3O2)O	lignin
0.119	Mangostin	CC(=CCC1=C(C2=C(C=C1O)OC3=C(C2=O)C(=C(C(=C3)O)OC)CC=C(C)C)O)C	lignin
0.119	(2S)-8-[(E)-3-hydroxy-3-methylbut-1-enyl]-5,7-dimethoxy-2-phenyl-2,3-dihydrochromen-4-one	CC(C)(/C=C/C1=C2C(=C(C=C1OC)OC)C(=O)C[C@H](O2)C3=CC=CC=C3)O	lignin
0.119	Orsellinic acid	CC1=CC(=CC(=C1C(=O)O)O)O	tannin

Mass (mg)	Name	SMILES	Classification
0.118	Glutamyl-S-(C3H5)-Cysteine sulfoxide	C/C=C/S(=O)CC(C(=O)O)NC(=O)CCC(C(=O)O)N	
0.118	FA 18:4+2O	O=C(O)CCCCCCCC(=O)C=CC=CC=CC(O)CC	lipid
0.118	MGMG 16:3	CC/C=C/C/C=C/C/C=C/CCCCCC(=O)OC(CO)COC1C(C(C(C(O1)CO)O)O)O	peptide
0.118	3-(8-hydroxyoctyl)phenol	C1=CC(=CC(=C1)O)CCCCCCCCO	lipid
0.117	2',6'-dihydroxy-4'-methoxydihydrochalcone	COC1=CC(=C(C(=C1)O)C(=O)CCC2=CC=CC=C2)O	lignin
0.117	FA 19:2	CCCCCCCCC/C=C/C=C/CCCCCC(=O)O	lipid
0.117	1,7-bis(4-hydroxyphenyl)heptane-3,5-diol	C1=CC(=CC=C1CCC(CC(CCC2=CC=C(C=C2)O)O)O)O	lignin
0.116	Cetraric acid	CCOCC1=C(C(=C(C2=C1OC(=O)C3=C(O2)C(=C(C=C3C)O)C=O)C)C(=O)O)O	tannin
0.116	NCGC00169633-02!9-phenyl-1-(2,4,6-trihydroxyphenyl)nonan-1-one	C1=CC=C(C=C1)CCCCCCCCC(=O)C2=C(C=C(C=C2O)O)O	
0.115	Tomelukast	CCCC1=C(C=CC(=C1O)C(=O)C)OCCCCC2=NNN=N2	lipid
0.115	Piceatannol	C1=CC(=C(C=C1/C=C/C2=CC(=CC(=C2)O)O)O)O	tannin
0.115	Cer 11:1;2O/2:0	CCCCCC/C=C/C(C(CO)NC(=O)C)O	lipid
0.115	methyl (4R,8aS)-1-hydroxy-2-(hydroxymethyl)-5,5,8a-trimethyl-4-[(2E,4E,6E)-octa-2,4,6-trienoyl]oxy-4a,6,7,8-tetrahydro-4H-naphthalene-1-carboxylate	C/C=C/C=C/C=C/C(=O)O[C@@H]1C=C(C([C@@]2(C1C(CCC2)(C)C)C)(C(=O)OC)O)CO	lipid
0.114	5-[6-(3-hydroxy-4-methoxyphenyl)-1,3,3a,4,6,6a-hexahydrofuro[3,4-c]furan-3-yl]-2-methoxyphenol	COC1=C(C=C(C=C1)C2C3COC(C3CO2)C4=CC(=C(C=C4)OC)O)O	lignin
0.114	Diisopropyl phthalate	CC(C)OC(=O)C1=CC=CC=C1C(=O)OC(C)C	lignin
0.114	Monobenzone	C1=CC=C(C=C1)COC2=CC=C(C=C2)O	soot
0.114	THYROTROPIN RELEASING HORMONE	C1C[C@H](N(C1)C(=O)[C@H](CC2=CN=CN2)NC(=O)[C@@H]3CCC(=O)N3)C(=O)N	lipid
0.114	Biochanin A	COC1=CC=C(C=C1)C2=COC3=CC(=CC(=C3C2=O)O)O	tannin
0.114	FA 18:5	CC/C=C/C=C/C=C/C=C/C=C/CCCCCC(=O)O	lipid
0.113	Hispidulin acetate	CC(=O)OC1=CC=C(C=C1)C2=CC(=O)C3=C(C(=C(C=C3O2)OC(=O)C)OC)OC(=O)C	tannin
0.113	Undecanedioic acid	C(CCCCC(=O)O)CCCCC(=O)O	lignin

S6. Predicted molecular properties related to different cytotoxic mechanisms for the organic molecules tentatively identified in the filter extract corresponding to the 80% cumulative concentration of organic compounds detected with LC-ESI/MS

Note that the molecules in Tables S6-S8 and Tables S9-S11 are listed in the same order.

Table S9. Molecular properties related to cytotoxicity for the organic molecules tentatively identified in the aqueous extract

ID	Name	LogP	Total ROS alerts	Total protein binding alerts
38	3-hydroxybenzoic acid	1.5	0	0
12	Benzene-1,2,4-triol	0.72	2	0
15	Idarubicin	2.34	0	0
52	p-Coumaric acid	1.46	0	2
42	2,3-Dihydroxybenzoic acid	1.2	1	0
51	3-PHENYLLACTIC ACID	0.78	0	0
137	Deuteromycol A	-0.11	1	0
93	UNPD123033	-3.17	0	1
390	Coniferylaldehyde	1.31	1	3
4	cis-4-Carboxymethylenebut-2-en-4-olide	0	0	2
35	Thymine	-0.62	0	0
293	Methylxanthoxylin	2.48	1	0
7	4-Thiouridine	-1.83	0	0
28	Vanillomandelic acid	0.54	1	0
246	4-Hydroxy-3-methoxycinnamaldehyde	1.31	1	3
16	1,3 Cyclohexanedione	0.46	0	0
33	Zonisamide	0.5	0	0
7	MANNITOL	-2.38	0	0
100	Alibendol	1.12	1	0
263	Azelaic acid (Not validated)	1.57	0	0
370	Heteropeucenin, Methyl Ether	3.83	1	0
64	Mandelic acid	0.62	0	0
11	Glutaric acid	-0.29	0	0
54	Acetovanillone;Apocynin	0.19	0	2
346	2',4-Dihydroxy-3,4',6'-Trimethoxychalcone	3.03	2	2

ID	Name	LogP	Total ROS alerts	Total protein binding alerts
150	4-hydroxycoumarin	1.03	0	0
402	Sinapoyl aldehyde	1.46	2	3
23	Methylxanthoxylin	2.48	1	0
49	Cumate	3.39	0	0
344	columbianetin	3.18	0	0
277	Syringaldehyde	0.73	1	0
31	Catechol	0.88	1	0
361	6-methoxy-7-(3-methylbut-2-enoxy)chromen-2-one	2.87	1	0
15	2-(1H-pyrrol-1-ylcarbonyl)benzene-1,3,5-triol	0.96	2	1
318	Acetosyringone	1.11	2	0
46	ethyl 2,3-dioxo-1,4-dihydroquinoline-4-carboxylate	0.42	0	0
119	vanillic acid	1.43	1	0
327	Chavicol O-beta-glucopyranoside	0.2	0	0
2	Succinic acid	-0.59	0	0
210	Vanillin	1.19	1	1
48	Orcinol	0.63	1	0
368	2-[(2R,8S,8aR)-8,8a-dimethyl-2,3,5,6,7,8-hexahydro-1H-naphthalen-2-yl]prop-2-enoic acid	4.28	1	0
230	6-Methoxy-7-hydroxycoumarin	1.89	0	2
332	1-(2,4-dihydroxyphenyl)-2-(3,5-dimethoxyphenyl)propan-1-one	3.05	1	0
89	Dehydrosalsolidine	2.28	1	0
348	7-Hydroxy-2',4',5'-trimethoxyisoflavan	3.76	2	0
373	Tetramisole	1.84	0	0
336	UNPD110798	1.69	0	0
244	6-Nitrobenzimidazole	1.48	1	0
445	no MS2: gamma-sitosterol	7.73	2	2
254	Sinapoyl aldehyde	1.46	0	2
352	Cumyluron	3.55	0	2
286	Umbelliferone	1.58	1	0

ID	Name	LogP	Total ROS alerts	Total protein binding alerts
333	Undecanedioic acid	2.8	0	0
354	Kanakugiol	3.02	0	0
331	Phloretin	2.74	0	0
365	5-O-Methylembelin	4.14	0	0
265	Boldenone_Undecylenate	7.42	2	3
167	Syringaldehyde	0.73	1	1
267	Agelasine (Stereochemistry Of Diterpene Unknown)	4.95	0	0
378	SB 205607	3.36	0	0
355	2-(2-hydroxypropan-2-yl)-6-(2-methylbut-3-en-2-yl)-2H,3H,7H-furo[3,2-g]chromen-7-one	4.16	0	0
13	2-methoxy-1,4-naphthoquinone	1.35	0	0
196	Corynoline	1.38	0	0
251	methyl 1-methyl-9H-beta-carbolin-7-yl ether	3.56	0	0
358	alpha,beta-Dihydroresveratrol	2.82	0	0
343	(1R,9S,10S)-3,4-dihydroxy-11,11-dimethyl-5-(propan-2-yl)-16-oxatetracyclo[7.5.2.0?,??.0?,?]hexadeca-2(7),3,5-triene-8,15-dione	2.68	0	0
374	methyl 2-ethyl-4-[(3R,4R,5S)-5-hydroxy-4,5-dimethyl-2-oxooxolan-3-yl]-2-methyl-3-oxobutanoate	2.26	0	0
272	NCGC00169706-02!6-ethyl-2,4-dihydroxy-3-methylbenzaldehyde	1.3	0	0
411	Triethylphosphate	0.8	0	0
325	8-Methylsulfinyloctyl isothiocyanate	4	0	0
457	Levetiracetam	-1.58	0	0
377	Tomelukast	3.14	0	0
340	Euparin	3.34	0	0
372	4-methoxy-9-(2-methylbut-3-en-2-yl)furo[3,2-g]chromen-7-one	3.44	0	0
330	Matairesinol	2.53	2	1
375	aurapten	4.82	0	0
180	Caffeic acid	1.15	0	0

ID	Name	LogP	Total ROS alerts	Total protein binding alerts
363	Isosakuranin	0.29	0	0
171	PYROGLUTAMATE	-0.59	0	0
342	Virensic acid	2.23	0	0
461	Angelic anhydride	2.67	0	0
170	SUBERIC ACID	0.59	0	0
337	4-Methyldaphnetin	0.5	0	0
190	Ethyl vanillin	1.59	0	0
190	Ethylparaben	2.46	0	0
338	2,3-Dihydroxybiphenyl	3.12	0	0
339	Cer 11:1;2O/2:0	1.01	0	0
31	ARABITOL	-2.58	0	0
40	Protocatechuic acid	0.86	0	0
128	1-(2-hydroxy-4,6-dimethoxyphenyl)ethanone	1.42	0	0
299	3-(3,4-Dimethoxyphenyl)-2-propenoic acid	2.34	0	0
97	Phthalic anhydride	0.68	0	0
397	5,7-Dimethoxy-4-methylcoumarin	1.81	0	0
260	Chlorogenoquinone (Not validated)	-0.51	0	0
57	Benzoic acid	1.87	0	0
19	ERYTHRITOL	-2.29	0	0
111	Gaultherin	-0.87	0	0

Table S10. Molecular properties related to cytotoxicity for the organic molecules tentatively identified in the methanol extract

ID	Name	LogP	ROS score	Protein binding score
6	Methylxanthoxylin	2.48	1	0
8	Salicylic acid	2.26	0	0
100	Boldenone_Undecylenate	7.42	0	2
222	Triethylphosphate	0.8	0	0
3	Pyrogallol	0.99	2	0

ID	Name	LogP	ROS score	Protein binding score
305	3,9-Dimethoxypterocarpan	3.31	1	0
322	SB 205607	3.36	0	0
16	p-Coumaric acid	1.46	0	2
281	2-[(2R,8S,8aR)-8,8a-dimethyl-2,3,5,6,7,8-hexahydro-1H-naphthalen-2-yl]prop-2-enoic acid	4.28	0	2
298	Heteropeucenin, Methyl Ether	3.83	1	0
334	Enterolactone	2.8	0	0
233	columbianetin	3.18	0	0
262	6-methoxy-7-(3-methylbut-2-enoxy)chromen-2-one	2.87	0	0
287	isoimperatorin	3.22	1	0
237	(??)-3',4'-Methylenedioxy-5,7-dimethylepicatechin	2.51	2	0
291	NCGC00187465-02!(E)-1-(5,7-dihydroxy-2,2,6-trimethylchromen-8-yl)-3-phenylprop-2-en-1-one	4.34	1	2
125	2,8-Phenazinediamine	2.39	0	0
211	1-(2,4-dihydroxyphenyl)-2-(3,5-dimethoxyphenyl)propan-1-one	3.05	1	0
207	m-Xylene-4-sulfonic acid	0.77	0	0
315	aurapten	4.82	0	0
311	adrenosterone	2.31	0	2
339	Kaempferol	1.86	1	0
255	2-(2-hydroxypropan-2-yl)-6-(2-methylbut-3-en-2-yl)-2H,3H,7H-furo[3,2-g]chromen-7-one	4.16	0	0
320	Tomelukast	3.14	0	2
337	angoletin	2.95	2	0
231	(1R,9S,10S)-3,4-dihydroxy-11,11-dimethyl-5-(propan-2-yl)-16-oxatetracyclo[7.5.2.0?,??.0?,?]hexadeca-2(7),3,5-triene-8,15-dione	2.48	0	0
220	UNPD110798	1.69	0	0
284	3-Hydroxy-3',4',5'-trimethoxyflavone	3.08	1	0
2	cis-4-Carboxymethylenebut-2-en-4-olide	0	1	0

ID	Name	LogP	ROS score	Protein binding score
228	(1,9,9b-trihydroxy-6,6,9a-trimethyl-3,5,5a,7,8,9-hexahydro-1H-benzo[e][2]benzofuran-5-yl) hexanoate	1.8	1	0
279	1-(4-methoxyphenyl)isoquinoline	3.86	1	0
330	Octadecanedioic acid	5.44	0	0
105	Agelasine (Stereochemistry Of Diterpene Unknown)	4.95	1	0
250	1,7-bis(3,4-dihydroxyphenyl)-6-hydroxyheptan-3-one	0.43	0	0
260	(2S)-Liquiritigenin	3	0	2
327	2-methoxy-5-methyl-3-(2-methylbut-3-en-2-yl)chromen-4-one	3.99	2	1
215	Phloretin	2.74	1	0
257	Dicyclohexyl phthalate	5.21	0	0
246	2-Hydroxyanthraquinone	3.05	1	0
222	Xanthone	3.39	2	0
290	Glutamyl-S-(C3H5)-Cysteine sulfoxide	-0.49	0	0
273	Isosakuranin	0.29	0	0
227	Virensic acid	2.23	1	3
247	MGMG 16:3	2.63	1	0
210	Coniferaldehyde	1.31	1	0
265	2,4-Dihydroxybenzophenone	2.65	0	0
264	Naproxen	3.18	1	0
213	ANGOLENSIN (R)	3.29	0	0
272	agnuside	0.09	0	0
276	FA 18:4+2O	3.29	0	0
232	Cetraric acid	2.1	0	0
318	NCGC00169633-02!9-phenyl-1-(2,4,6-trihydroxyphenyl)nonan-1-one	3.93	0	0
253	Methyl 1-naphthaleneacetate	3.5	0	0
214	5-[6-(3-hydroxy-4-methoxyphenyl)-1,3,3a,4,6,6a-hexahydrofuro[3,4-c]furan-3-yl]-2-methoxyphenol	2.6	1	0
306	Biochanin A	2.78	1	0

ID	Name	LogP	ROS score	Protein binding score
248	Piceatannol	2.93	0	0
216	Undecanedioic acid	2.8	0	0
278	3-(8-hydroxyoctyl)phenol	3.46	2	1
223	2,3-Dihydroxybiphenyl	3.12	0	0
282	FA 19:2	7.36	0	0
225	Cer 8:1;2O/5:0	0.19	0	0
234	N-acetyltryptophan	-1.69	0	0

Table S11. Molecular properties related to cytotoxicity for the organic molecules tentatively identified in the acetone extract

ID	Name	LogP	ROS score	Protein binding score
3	Methylxanthoxylin	2.48	1	0
79	Boldenone_Undecylenate	7.42	0	2
176	Triethylphosphate	0.8	0	0
4	Salicylic acid	2.26	0	0
227	3,9-Dimethoxypterocarpan	3.31	1	0
246	11-Dehydrocorticosterone	2.55	0	2
191	2-[(2R,8S,8aR)-8,8a-dimethyl-2,3,5,6,7,8-hexahydro-1H-naphthalen-2-yl]prop-2-enoic acid	4.28	0	2
218	gamma-sitosterol	7.73	0	0
10	Acetovanillone;Apocynin	0.19	0	2
2	Pyrogallol	0.99	2	0
257	Enterolactone	2.8	0	0
218	Heteropeucenin, Methyl Ether	3.83	0	2
9	p-Coumaric acid	1.46	1	0
151	columbianetin	3.18	0	0
178	6-methoxy-7-(3-methylbut-2-enoxy)chromen-2-one	2.87	1	0
205	isoimperatorin	3.22	0	0
154	(??)-3',4'-Methylenedioxy-5,7-dimethylepicatechin	2.51	2	0

ID	Name	LogP	ROS score	Protein binding score
240	aurapten	4.82	0	0
208	NCGC00187465-02!(E)-1-(5,7-dihydroxy-2,2,6-trimethylchromen-8-yl)-3-phenylprop-2-en-1-one	4.34	0	0
233	2-Methoxyestrone	3.28	1	0
132	1-(2,6-dihydroxy-4-methoxyphenyl)-3-(4-methoxyphenyl)propan-1-one	3.06	0	0
225	4-Hydroxy-2',4',6'-trimethoxychalcone	3.33	1	0
172	2-(2-hydroxypropan-2-yl)-6-(2-methylbut-3-en-2-yl)-2H,3H,7H-furo[3,2-g]chromen-7-one	4.16	1	0
261	angoletin	2.95	1	2
6	4-HYDROXYBENZOATE	1.58	1	0
262	Kaempferol	1.86	1	2
150	Malabaricano	4.62	1	0
156	ANGOLENSIN (R)	3.29	1	0
254	Octadecanedioic acid	5.44	0	0
146	(1,9,9b-trihydroxy-6,6,9a-trimethyl-3,5,5a,7,8,9-hexahydro-1H-benzo[e][2]benzofuran-5-yl) hexanoate	1.8	0	0
177	NCGC00385014-01!(E)-1,7-bis(4-hydroxyphenyl)hept-1-en-3-one	4.09	0	0
171	Kanakugiol	3.02	0	0
174	Dicyclohexyl phthalate	5.21	0	0
136	Phloretin	2.74	0	0
141	1,9-dimethyluric acid	-0.78	2	0
170	Isoflavone base + 3O, 1Prenyl	4	2	1
176	(2S)-Liquiritigenin	3	1	0
256	Decursin	3.85	1	0
163	2-Hydroxyanthraquinone	3.05	2	0
202	3-Hydroxy-3',4',5'-trimethoxyflavone	3.08	1	0
220	Mangostin	5.36	0	2
238	(2S)-8-[(E)-3-hydroxy-3-methylbut-1-enyl]-5,7-dimethoxy-2-phenyl-2,3-dihydrochromen-4-one	5.37	2	0

ID	Name	LogP	ROS score	Protein binding score
147	Orsellinic acid	1.6	0	0
207	Glutamyl-S-(C3H5)-Cysteine sulfoxide	-0.49	1	0
190	FA 18:4+2O	3.29	1	0
162	MGMG 16:3	2.63	0	0
197	3-(8-hydroxyoctyl)phenol	3.46	0	0
133	2',6'-dihydroxy-4'-methoxydihydrochalcone	3.21	2	2
200	FA 19:2	7.36	0	0
236	1,7-bis(4-hydroxyphenyl)heptane-3,5-diol	4.3	1	0
149	Cetraric acid	2.1	0	0
244	NCGC00169633-02!9-phenyl-1-(2,4,6-trihydroxyphenyl)nonan-1-one	3.93	1	0
245	Tomelukast	3.14	1	0
164	Piceatannol	2.93	0	2
144	Cer 11:1;2O/2:0	1.01	0	0
222	methyl (4R,8aS)-1-hydroxy-2-(hydroxymethyl)-5,5,8a-trimethyl-4-[(2E,4E,6E)-octa-2,4,6-trienoyl]oxy-4a,6,7,8-tetrahydro-4H-naphthalene-1-carboxylate	3.61	0	0
134	5-[6-(3-hydroxy-4-methoxyphenyl)-1,3,3a,4,6,6a-hexahydrofuro[3,4-c]furan-3-yl]-2-methoxyphenol	2.6	0	0
180	Diisopropyl phthalate	2.83	0	0
169	Monobenzone	3.2	0	0
193	THYROTROPIN RELEASING HORMONE	-2.46	0	0
228	Biochanin A	2.78	0	0
221	FA 18:5	5.67	0	0
223	Hispidulin acetate	2.1	1	0
137	Undecanedioic acid	2.8	0	0

Table S12. LogP-score values, individual and cumulative percentage contributions, and SMILES of the tentatively identified molecules.

LogP score			Name	SMILES
Value	%	Cumulative		
129.0	9%	9%	3-hydroxybenzoic acid	C1=CC(=CC(=C1)O)C(=O)O
85.4	6%	15%	Idarubicin	C1=CC=C2C(=C1)C(=C(N2)O)C3=NC4=CC=CC=C4C3=O
59.1	4%	19%	Boldenone_Undecylenate	CC12CC[C@H]3C([C@@H]1CC[C@@H]2OC(=O)CCCCCCCCC=C)CCC4=CC(=O)C=CC34C
48.7	3%	23%	p-Coumaric acid	C1=CC(=CC=C1/C=C/C(=O)O)O
37.8	3%	26%	Benzene-1,2,4-triol	OC1=CC=C(O)C(O)=C1
37.1	3%	28%	Heteropeucenin,Methyl Ether	CC1=CC(=O)C2=C(O1)C(=C(C=C2O)OC)CC=C(C)C
28.9	2%	30%	No MS2: gamma-sitosterol	CC[C@@H](CC[C@@H](C)[C@H]1CC[C@@H]2[C@@]1(CC[C@H]3[C@H]2CC=C4[C@@]3(CC[C@@H](C4)O)C)C)C(C)C
28.9	2%	32%	2,3-Dihydroxybenzoic acid	C1=CC(=C(C(=C1)O)O)C(=O)O
24.8	2%	34%	2',4 Dihydroxy-3,4',6'-Trimethoxychalcone	COC1=CC(=C(C(=C1)OC)C(=O)/C=C/C2=CC(=C(C=C2)O)OC)O
23.6	2%	36%	Cumate	CC(C)C1=CC=C(C=C1)C(=O)[O-]
22.0	2%	37%	columbianetin	CC(C)([C@@H]1CC2=C(O1)C=CC3=C2OC(=O)C=C3)O
20.4	1%	40%	Coniferylaldehyde	COC1=CC(\C=C\C=O)=CC=C1O
19.6	1%	42%	2-[(2R,8S,8aR)-8,8a-dimethyl-2,3,5,6,7,8-hexahydro-1H-naphthalen-2-yl]prop-2-enoic acid	C[C@H]1CCCC2=CC[C@H](C[C@]12C)C(=C)C(=O)O

LogP score			Name	SMILES
Value	%	Cumulative		
18.9	1%	43%	6-methoxy-7-(3-methylbut-2-enoxy)chromen-2-one	CC(=CCOC1=C(C=C2C=CC(=O)OC2=C1)OC)C
18.6	1%	44%	Methylxanthoxylin	O=C(C=1C(O)=C(C(OC)=CC1OC)C)C
17.3	1%	46%	3,9-Dimethoxypterocarpan	COC1=CC2=C(C=C1)[C@@H]3COC4=C([C@@H]3O2)C=CC(=C4)OC
16.4	1%	47%	SB 205607	CN1CC[C@@]2(CC3=NC4=CC=CC=C4C=C3C[C@H]2C1)C5=CC(=CC=C5)O.O.Br
15.9	1%	49%	Agelasine (Stereochemistry Of Diterpene Unknown)	
14.9	1%	50%	7-Hydroxy-2',4',5'-trimethoxyisoflavan	COC1=CC(=C(C=C1C2CC3=C(C=C(C=C3)O)OC2)OC)OC
14.6	1%	51%	3-PHENYLLACTIC ACID	C1=CC=C(C=C1)CC(C(=O)O)O
13.7	1%	53%	5-O-Methylembelin	CCCCCCCCCCCC1=C(C(=O)C=C(C1=O)OC)O
13.2	1%	54%	aurapten	CC(=CCC/C(=C/COC1=CC2=C(C=C1)C=CC(=O)O2)/C)C
13.0	1%	55%	1-(2,4-dihydroxyphenyl)-2-(3,5-dimethoxyphenyl)propan-1-one	CC(C1=CC(=CC(=C1)OC)OC)C(=O)C2=C(C=C(C=C2)O)O
13.0	1%	56%	2-(2-hydroxypropan-2-yl)-6-(2-methylbut-3-en-2-yl)-2H,3H,7H-furo[3,2-g]chromen-7-one	
12.8	1%	57%	Dicyclohexyl phthalate	C1CCC(CC1)OC(=O)C2=CC=CC=C2C(=O)OC3CCCCC3
12.7	1%	58%	Cumyluron	CC(C)(C1=CC=CC=C1)NC(=O)NCC2=CC=CC=C2Cl

LogP score			Name	SMILES
Value	%	Cumulative		
12.5	1%	59%	NCGC00187465-02!(E)-1-(5,7-dihydroxy-2,2,6-trimethylchromen-8-yl)-3-phenylprop-2-en-1-one	CC1=C(C2=C(C(=C1O)C(=O)/C=C/C3=CC=CC=C3)OC(C=C2)(C)C)O
11.6	1%	60%	8-Methylsulfinyloctyl isothiocyanate	
11.2	1%	61%	Alibendol	C1=C(C(=C(C=C1CC=C)C(=O)NCCO)O)OC
10.8	1%	64%	methyl 1-methyl-9H-beta-carbolin-7-yl ether	
10.5	1%	64%	isoimperatorin	CC(=CCOC1=C2C=CC(=O)OC2=CC3=C1C=CO3)C
10.3	1%	65%	Kanakugiol	COC1=C(C(=C(C(=C1C(=O)/C=C/C2=CC=CC=C2)O)OC)OC)OC
10.2	1%	66%	Enterolactone	C1[C@H]([C@@H](C(=O)O1)CC2=CC(=CC=C2)O)CC3=CC(=CC=C3)O
9.8	1%	66%	2-methoxy-5-methyl-3-(2-methylbut-3-en-2-yl)chromen-4-one	CC1=C2C(=CC=C1)OC(=C(C2=O)C(C)(C)C=C)OC
9.6	1%	67%	1-(4-methoxyphenyl)isoquinoline	COC1=CC=C(C=C1)C2=NC=CC3=CC=CC=C32
9.5	1%	68%	4-methoxy-9-(2-methylbut-3-en-2-yl)furo[3,2-g]chromen-7-one	
9.3	1%	69%	Phloretin	C1=CC(=CC=C1CCC(=O)C2=C(C=C(C=C2O)O)O)O
9.3	1%	70%	Euparin	
9.2	1%	70%	NCGC00169633-02!9-phenyl-1-(2,4,6-trihydroxyphenyl)nonan-1-one	C1=CC=C(C=C1)CCCCCCCCC(=O)C2=C(C=C(C=C2O)O)O

LogP score			Name	SMILES
Value	%	Cumulative		
9.0	1%	71%	Dehydrosalsolidine	CC1=NCCC2=CC(=C(C=C12)OC)OC
8.9	1%	72%	Tomelukast	
8.4	1%	72%	alpha,beta-Dihydroresveratrol	
8.4	1%	73%	6-Methoxy-7-hydroxycoumarin	COC1=C(C=C2C(=C1)C=CC(=O)O2)O
8.2	1%	74%	Xanthone	C1=CC=C2C(=C1)C(=O)C3=CC=CC=C3O2
8.1	1%	75%	Methyl 1-naphthaleneacetate	COC(=O)CC1=CC=CC2=CC=CC=C21
8.0	1%	75%	(1R,9S,10S)-3,4-dihydroxy-11,11-dimethyl-5-(propan-2-yl)-16-oxatetracyclo[7.5.2.0?,??.0?,?]hexadeca-2(7),3,5-triene-8,15-dione	
8.0	1%	76%	2,3-Dihydroxybiphenyl	
8.0	1%	76%	3-(8-hydroxyoctyl)phenol	C1=CC(=CC(=C1)O)CCCCCCCCO
7.9	1%	77%	4-hydroxycoumarin	O=C1OC=2C=CC=CC2C(O)=C1
7.8	1%	77%	ANGOLENSIN (R)	C[C@H](C1=CC=C(C=C1)OC)C(=O)C2=C(C=C(C=C2)O)O
7.8	1%	78%	3-Hydroxy-3',4',5'-trimethoxyflavone	COC1=CC(=CC(=C1OC)OC)C2=C(C(=O)C3=CC=CC=C3O2)O
7.8	1%	79%	FA 18:4+2O	O=C(O)CCCCCCCC(=O)C=CC=CC=CC(O)CC
7.7	1%	79%	vanillic acid	O=C(O)C1=CC=C(O)C(OC)=C1

LogP score			Name	SMILES
Value	%	Cumulative		
7.6	1%	80%	(??)-3',4'-Methylenedioxy-5,7-dimethylepicatechin	OC1CC=2C(OC)=CC(OC)=CC2OC1C3=CC=C4OCOC4=C3
7.6	1%	80%	angoletin	CC1=C(C(=C(C(=C1O)C(=O)CCC2=CC=CC=C2)OC)C)O
7.4	1%	81%	2-Hydroxyanthraquinone	C1=CC=C2C(=C1)C(=O)C3=C(C2=O)C=C(C=C3)O
7.4	1%	81%	(2S)-Liquiritigenin	C1[C@H](OC2=C(C1=O)C=CC(=C2)O)C3=CC=C(C=C3)O
7.2	1%	82%	Tetramisole	C1CSC2=NC(CN21)C3=CC=CC=C3
7.1	1%	83%	Angelic anhydride	

S7. Results of the statistical analyses of MTT and LDH results

Table S13. Statistical analysis of the MTT data obtained from Dunnett's multiple comparisons test using two-way ANOVA for the three extraction solvents.

BrC concentrations ($\mu g\ mL^{-1}$)	Significance/extraction solvent	Adjusted *p*-value
**Water**		
0 (control) vs. 37	Yes	0.0256
0 vs. 75	Yes	0.0059
0 vs. 112	Yes	<0.0001
0 vs. 150	Yes	<0.0001
0 vs. 300	Yes	<0.0001
0 vs. 600	Yes	<0.0001
**MeOH**		
0 (control) vs. 37	Yes	<0.0001
0 vs. 75	Yes	<0.0001
0 vs. 112	Yes	<0.0001
0 vs. 150	Yes	<0.0001
0 vs. 300	Yes	<0.0001
0 vs. 600	Yes	<0.0001
**Acetone**		
0 (control) vs. 37	Yes	<0.0001
0 vs. 75	Yes	<0.0001
0 vs. 112	Yes	<0.0001
0 vs. 150	Yes	<0.0001
0 vs. 300	Yes	<0.0001
0 vs. 600	Yes	<0.0001

Table S14. Statistical analysis of the MTT data obtained from Tukey's multiple comparisons test using two-way ANOVA analysis for the three extraction solvents.

**Extraction solvents**	**Significance/BrC concentration (µg mL^{-1})**	**Adjusted *p*-value**
**Control**		
MeOH vs. Acetone	No	0.4321
MeOH vs. Water	No	0.9938
Acetone vs. Water	No	0.5788
**37**		
MeOH vs. Acetone	Yes	0.0038
MeOH vs. Water	Yes	<0.0001
Acetone vs. Water	Yes	<0.0001
**75**		
MeOH vs. Acetone	Yes	<0.0001
MeOH vs. Water	Yes	<0.0001
Acetone vs. Water	Yes	<0.0001
**112**		
MeOH vs. Acetone	Yes	<0.0001
MeOH vs. Water	Yes	<0.0001
Acetone vs. Water	Yes	<0.0001
**150**		
MeOH vs. Acetone	No	0.1126
MeOH vs. Water	Yes	<0.0001
Acetone vs. Water	Yes	<0.0001

**Extraction solvents**	**Significance/BrC concentration (µg mL^{-1})**	**Adjusted *p*-value**
**300**		
MeOH vs. Acetone	Yes	0.0127
MeOH vs. Water	Yes	<0.0001
Acetone vs. Water	Yes	<0.0001
**600**		
MeOH vs. Acetone	No	0.0945
MeOH vs. Water	No	0.8822
Acetone vs. Water	No	0.0777

Table S15. Statistical analysis of the LDH data obtained from Dunnett's multiple comparisons test using two-way ANOVA analysis for the three extraction solvents.

**BrC concentrations (µg mL^{-1})**	**Significance/extraction solvent**	**Adjusted *p*-value**
**Water**		
0 (control) vs. 37	No	0.4504
0 vs. 75	No	0.4268
0 vs. 112	No	0.5852
0 vs. 150	No	0.4591
0 vs. 300	No	0.1772
0 vs. 600	Yes	0.0017
**MeOH**		
0 (control) vs. 37	No	0.7375

**BrC concentrations (μg mL^{-1})**	**Significance/extraction solvent**	**Adjusted *p*-value**
0 vs. 75	No	0.3173
0 vs. 112	No	0.8079
0 vs. 150	No	0.3038
0 vs. 300	Yes	<0.0001
0 vs. 600	Yes	<0.0001
**Acetone**		
0 (control) vs. 37	No	0.3653
0 vs. 75	No	0.0516
0 vs. 112	No	0.1153
0 vs. 150	Yes	<0.0001
0 vs. 300	Yes	<0.0001
0 vs. 600	Yes	<0.0001

Table S16. Statistical analysis of the LDH data obtained from Tukey's multiple comparisons test using two-way ANOVA analysis across all fractions.

**Extraction solvents**	**Significance/BrC concentration (μg mL^{-1})**	**Adjusted p-value**
**Control**		
Acetone vs. MeOH	No	>0.9999
Acetone vs. Water	No	>0.9999
MeOH vs. Water	No	>0.9999
**37**		

Extraction solvents	Significance/BrC concentration (μg mL^{-1})	Adjusted p-value
Acetone vs. MeOH	No	0.8742
Acetone vs. Water	No	0.9992
MeOH vs. Water	No	0.8917
	**75**	
Acetone vs. MeOH	No	0.7186
Acetone vs. Water	No	0.6234
MeOH vs. Water	No	0.9873
	**112**	
Acetone vs. MeOH	No	0.4548
Acetone vs. Water	No	0.6809
MeOH vs. Water	No	0.9301
	**150**	
Acetone vs. MeOH	Yes	<0.0001
Acetone vs. Water	Yes	<0.0001
MeOH vs. Water	No	0.9711
	**300**	
Acetone vs. MeOH	Yes	<0.0001
Acetone vs. Water	Yes	<0.0001
MeOH vs. Water	Yes	<0.0001
	**600**	

Extraction solvents	Significance/BrC concentration (µg mL^{-1})	Adjusted p-value
Acetone vs. MeOH	Yes	<0.0001
Acetone vs. Water	Yes	<0.0001
MeOH vs. Water	Yes	<0.0001

References

(1) MoNA. *https://mona.fiehnlab.ucdavis.edu/downloads*. MassBank of North America, 2024. (accessed Aug 08 2025).

(2) MS-DIAL. *https://systemsomicslab.github.io/compms/msdial/main.html#MSP*. 2024. (accessed Aug 08 2025).

(3) Vaniya, A.; Samra, S. N.; Palazoglu, M.; Tsugawa, H.; Fiehn, O. Using MS-FINDER for identifying 19 natural products in the CASMI 2016 contest. *Phytochem Lett* **2017**, *21*, 306-312. DOI: https://doi.org/10.1016/j.phytol.2016.12.008.

(4) Koch, B. P.; Dittmar, T. From mass to structure: an aromaticity index for high-resolution mass data of natural organic matter. *Rapid Commun. Mass Spectrom.* **2006**, *20* (5), 926-932. DOI: https://doi.org/10.1002/rcm.2386.

(5) Moschos, V.; Christensen, C.; Mouton, M.; Fiddler, M. N.; Isolabella, T.; Mazzei, F.; Massabò, D.; Turpin, B. J.; Bililign, S.; Surratt, J. D. Quantifying the Light-Absorption Properties and Molecular Composition of Brown Carbon Aerosol from Sub-Saharan African Biomass Combustion. *Environ. Sci. Technol.* **2024**, *58* (9), 4268-4280. DOI: https://doi.org/10.1021/acs.est.3c09378.

(6) Hughey, C. A.; Hendrickson, C. L.; Rodgers, R. P.; Marshall, A. G.; Qian, K. Kendrick Mass Defect Spectrum: A Compact Visual Analysis for Ultrahigh-Resolution Broadband Mass Spectra. *Anal. Chem.* **2001**, *73* (19), 4676-4681. DOI: https://doi.org/10.1021/ac010560w.

(7) Kroll, J. H.; Donahue, N. M.; Jimenez, J. L.; Kessler, S. H.; Canagaratna, M. R.; Wilson, K. R.; Altieri, K. E.; Mazzoleni, L. R.; Wozniak, A. S.; Bluhm, H. Carbon oxidation state as a metric for describing the chemistry of atmospheric organic aerosol. *Nat. Chem.* **2011**, *3* (2), 133-139. DOI: https://doi.org/10.1038/nchem.948.

(8) Laszakovits, J. R.; MacKay, A. A. Data-based chemical class regions for Van Krevelen diagrams. *J. Am. Soc. Mass Spectrom.* **2021**, *33* (1), 198-202. DOI: https://doi.org/10.1021/jasms.1c00230.

(9) Bhatia, M. P.; Das, S. B.; Longnecker, K.; Charette, M. A.; Kujawinski, E. B. Molecular characterization of dissolved organic matter associated with the Greenland ice sheet. *Geochim. Cosmochim. Acta* **2010**, *74* (13), 3768-3784. DOI: https://doi.org/10.1016/j.gca.2010.03.035.
